# A non‐surgical method for subretinal delivery by trans‐scleral microneedle injection

**DOI:** 10.1002/btm2.10755

**Published:** 2025-01-28

**Authors:** Amir Hejri, Micah A. Chrenek, Nolan T. Goehring, Isabella I. Bowland, Richard Noel, Jiong Yan, John M. Nickerson, Mark R. Prausnitz

**Affiliations:** ^1^ School of Chemical and Biomolecular Engineering Georgia Institute of Technology Atlanta Georgia USA; ^2^ Department of Ophthalmology Emory University Atlanta Georgia USA; ^3^ Department of Animal Resources Georgia Institute of Technology Atlanta Georgia USA

**Keywords:** ocular drug delivery, posterior segment, subretinal, transscleral subretinal injection microneedle injector

## Abstract

Novel therapeutics have emerged for treating neurodegenerative eye diseases but are limited by non‐optimal methods of ocular administration. Subretinal injection is the preferred method of delivery for retinal gene and stem‐cell therapies, but its invasive and complex surgical procedure is a major limiting factor in clinical investigations and practice. Here, we engineered a novel trans‐scleral injection technique to safely administer to the subretinal space in a simple, non‐surgical, and minimally invasive procedure. Subretinal injection using this technique in rodents and rabbits took <1 min per injection and did not require a surgical microscope. Extensive safety examinations in rats showed that the injection technique reliably administered into the subretinal space with no incidence of retinal perforation, little or no choroidal bleeding, and no evidence of retinal toxicity. We further found that repeated subretinal injection in the same eye, in rats, was well tolerated. The developed technique may enable non‐surgical subretinal injection without vitrectomy, potentially increasing safety, efficacy, and access to ocular therapies.

**Translational Impact Statement**

This work addresses a key unmet need in retinal delivery by improving the current subretinal injection method, which is complex and clinically limiting. Here, a robust, minimally invasive technique was developed and tested preclinically, laying the groundwork for a novel method of subretinal delivery that could eliminate the need for vitrectomy in humans and potentially be performed as an office procedure.

## 1 INTRODUCTION

Many powerful new therapies have been developed for eye disorders, including gene therapy with viral vectors and regenerative medicine using stem cells, some of which have made it to clinical investigation.^1^, ^2^, ^3^ Notably, the first Food and Drug Administration‐approved gene therapy product is a treatment for a genetic ocular condition (i.e., biallelic RPE65 mutation‐associated retinal dystrophy).^4^, ^5^ These next‐generation therapies have great promise in treating previously incurable blinding eye disorders but are limited by delivery methods that can achieve safety and efficacy.^4^, ^6^, ^7^, ^8^, ^9^ These therapies generally require injection into the subretinal space (SRS), which can be a barrier to clinical translation and widespread adoption.^10^

In subretinal injection, materials are delivered into the SRS, a potential space separating neural retina from the underlying retinal pigment epithelium (RPE), which expands to form a bleb upon fluid injection. Subretinal delivery is an ideal route of administration for gene and cell therapies because (1) it enables highly targeted administration adjacent to the retina and RPE cells,^2^ thereby (2) requiring lower drug dose to achieve a therapeutic effect, and (3) minimizing off‐target effects, (4) it bypasses the major drug barriers of the eye such as inner limiting membrane (ILM) of the retina and the blood retina barrier, and (5) intact SRS is an immune‐privileged site that lowers the risk of systemic exposure, inflammatory response, immune rejection, and neutralization by humoral antibodies, a feature critical to the success of gene and cell therapies.^11^, ^12^ These unique benefits, however, are overshadowed by the complex, poorly controlled and costly surgical procedure of subretinal delivery that has raised safety concerns and limited patient access.^6^, ^10^

The subretinal injection procedure conventionally involves a three‐port pars plana vitrectomy surgery followed by transvitreal insertion of a thin needle (e.g., 33–41 G) across the vitreous cavity and into the SRS by making a small perforation in the neural retina (i.e., retinotomy).^13^ There are multiple challenges associated with this approach. First, vitrectomy carries a risk of cataract formation, eye infection (i.e., endophthalmitis) and retinal detachment.^14^ Second, the retinotomy introduces a full‐thickness retinal break that can lead to chronic retinal detachment, macular hole, and permanent vision loss.^15^, ^16^, ^17^, ^18^, ^19^ Furthermore, the retinotomy enables a pathway out of the SRS and into the vitreous, resulting in fluid reflux that is frequently and inevitably seen during subretinal injection.^9^

Vitreous reflux has many drawbacks. It causes inaccurate subretinal dosing which compromises treatment efficacy and makes clear assessment of treatment outcomes difficult.^9^ In some cases, it can even lead to failed injection attempt due to inability to raise a subretinal bleb.^10^ Moreover, vitreous reflux increases systemic exposure because vitreous humor has a lower immune‐privileged protection compared to the SRS.^8^ In cell delivery, leaked cells could proliferate in the vitreous leading to epiretinal membrane formation, retinal detachment, and proliferative vitreoretinopathy.^9^

As an additional limitation, current subretinal injection procedures are difficult to control. Needle tip misplacement is possible because there is no inherent mechanism to control penetration depth once the needle tip is in the retina. Shallow penetration causes drug loss by injection into the vitreous or intraretinally,^10^ while penetrating too deep disrupts RPE and choroidal hemorrhage is possible.^20^ As a result, injection success relies heavily on surgical skill, which necessitates access to a specifically trained and experienced surgeon. Lastly, given the invasive and risky nature of the procedure, repeated subretinal injections are difficult and generally contraindicated.^6^, ^7^, ^8^, ^10^, ^18^ Although gene and stem cell therapies are designed for long‐lasting effects, their efficacy may wane over time and eventually require re‐administration to boost or renew the therapeutic effect.

Intravitreal and suprachoroidal space (SCS) injections are alternative routes of administration with less‐invasive and much‐simpler procedures compared to subretinal injection but have had limited success in retinal delivery due to their lack of retinal targeting. The bioavailability of intravitreally delivered drugs in the retina is reduced by the presence of the ILM that hinders diffusion of molecules, virus particles, and cells from vitreous into the retina.^2^ Suprachoroidally administered drugs, on the other hand, must diffuse across the choroid, Bruch's membrane and RPE tight junctions while escaping clearance by the highly vascular choriocapillaris before reaching the retina.^18^, ^21^, ^22^ Additionally, both delivery methods suffer from higher systemic exposure due to less immune‐privileged status compared to their subretinal injection counterparts which increases the risk of off‐target effects, inflammatory immune reaction, and rejection in gene and cell therapies.^11^

Given these challenges, an ideal method of subretinal delivery should be (1) simple to perform without surgical training (e.g., as an office procedure), (2) targeted without vitreous reflux to enable accurate dosing and minimize systemic exposure, (3) minimally invasive (i.e., avoiding the need for vitrectomy or retinotomy), and (4) low cost to increase patient access.

Here, we propose a new method of subretinal delivery involving a trans‐scleral microneedle (MN) injection that is designed to achieve the characteristics of an ideal subretinal delivery method. In this approach, a hollow MN is inserted into the eye and injects into the SRS by penetrating across sclera, choroid, and RPE without requiring vitrectomy or retinotomy. MNs are miniaturized needles typically measuring <1 mm in length, which can be controlled to enable precise penetration into tissue, as shown by their successful use for suprachoroidal injections.^23^

Previous attempts to access the SRS through trans‐scleral insertion using hypodermic needles have raised concerns over safety and reliability, including the risk of significant (and potentially unstoppable) choroidal hemorrhage^18^, ^24^, ^25^, ^26^, ^27^, ^28^ and retinal perforation from uncontrolled needle penetration.^13^, ^29^ As a result, trans‐scleral SRS injection has been limited largely to use in animal research with almost no use in clinical practice. In contrast, we propose that the use of much shorter and thinner MNs of optimal length to target SRS, which enable highly controlled trans‐scleral and trans‐choroidal penetration and are designed to minimize or eliminate choroidal bleeding and retinal perforation. This approach can facilitate precise and targeted dosing to the SRS without drug loss into the vitreous, offering a simple and minimally invasive technique that does not require vitrectomy and holds promise for further development in clinical applications.

## 2 RESULTS

### 2.1 Microneedle designed to minimize choroidal bleeding after trans‐scleral insertion

While it is recognized that trans‐scleral injection into the SRS has many advantages, its development for clinical application has been limited by the expectation that choroidal puncture bears a risk of uncontrolled hemorrhage that should be avoided given its known toxicity to the retina.^15^, ^24^, ^25^, ^26^, ^27^, ^30^ We hypothesize that the extent of choroidal bleeding is proportional to the degree of damage to choroidal vasculature, which is also proportional to needle size. This means that hemorrhage could be reduced or eliminated by using a needle small enough to minimize rupture of choroidal vessels. As a guide to our experimental design, small penetrating electrodes (90–100 μm) in trans‐scleral, trans‐choroidal retinal stimulation studies in rabbits demonstrated no choroidal hemorrhage during insertion and a strong correlation between adverse effects and electrode size when using electrodes of larger diameter (e.g., 350 μm).^30^, ^31^, ^32^ Thus, in our experiments we posited that microneedles of similar diameter (~100 μm) should be sufficiently small to avoid vascular damage, while larger needles would more likely cause hemorrhage.

**FIGURE 1:**
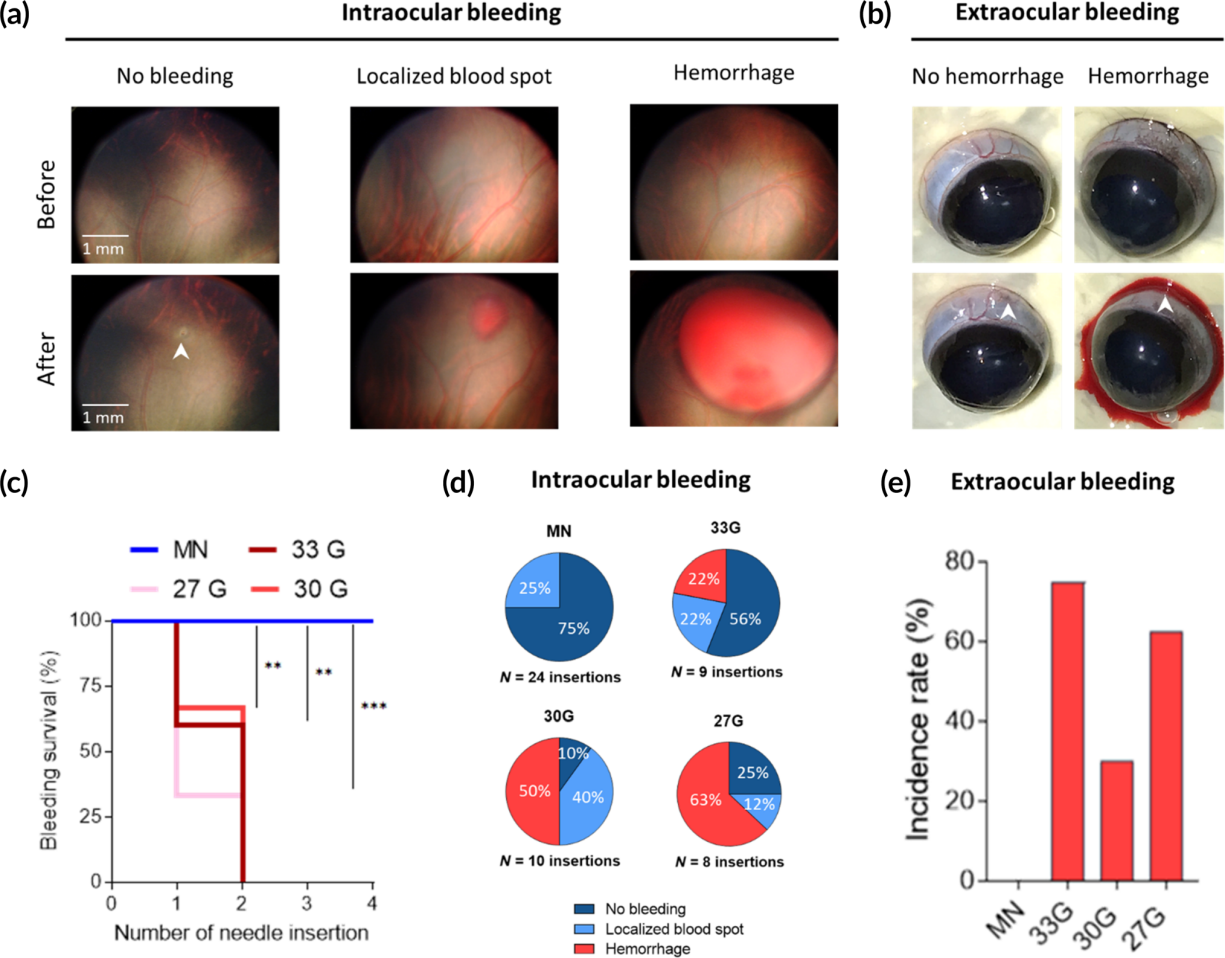
Qualitative and quantitative assessment of the effect of needle size on ocular bleeding after trans‐scleral insertion into the subretinal space of rats in vivo. Representative brightfield fundus (a) and ocular surface (b) images taken before and immediately after needle insertion showing various bleeding outcomes. (c) A “survival curve” showing incidence of intraocular or extraocular hemorrhage after needle insertion. Each needle size was tested in *n* = 6 eyes with each eye receiving up to four insertions unless hemorrhage occurred. The total study included *n* = 24 eyes in *n* = 12 rats. Frequency of intraocular (d) and extraocular (e) bleeding outcomes as a function of needle size. Hemorrhage survival rates across needles sizes shown in (c) were compared using a Log‐rank (Mantel‐Cox) statistical test.

To test this hypothesis, we assessed the incidence of ocular bleeding following insertion of needles of various sizes across the sclera and choroid of the rat eye in vivo (Figure 1). We repeatedly inserted a needle up to four times per eye and found that all eyes (18/18) into which hypodermic needles with outer diameter (OD) of 210 μm (33 G), 310 μm (30 G) or 410 μm (27 G) were inserted experienced intraocular and/or extraocular hemorrhage after just one or two insertions (Figure 1c). In contrast, among eyes that received insertion of a MN with OD of 110 μm, there were no eyes (0/6) exhibiting intraocular or extraocular hemorrhage, even after four sequential insertions per eye (i.e., 24 total insertions). This finding shows that the small size of the MN avoided ocular hemorrhage even after repeated MN insertions, while hypodermic needles led to hemorrhage in all cases.

In addition to the cumulative effect of multiple insertions, we also considered the incidence of hemorrhage after each individual needle insertion and found that hypodermic needles had a 22%–63% chance of intraocular hemorrhage per insertion (Figure 1d) and a 30%–75% chance of extraocular hemorrhage per insertion (Figure 1b,e). In contrast, we never observed intraocular or extraocular hemorrhage in eye punctures with MNs (Figure 1d,e). Furthermore, MN insertion caused no evidence of blood at all in 75% (18/24) of ocular insertions (“No bleeding” in Figure 1a), or a highly localized, self‐limited blood spot (≤1 mm^2^) in the remaining 25% (6/24) of eyes (Figure 1d), none of which are expected to be of clinical significance.^33^, ^34^, ^35^

### 2.2 Subretinal delivery by trans‐scleral microneedle injection

Subretinal injection involves not only crossing the choroid without hemorrhage but also precisely placing the needle tip within the SRS without deeper penetration into neural retina. Having reduced needle width to avoid hemorrhage, we next controlled needle penetration depth by (1) optimizing MN length to penetrate sclera and choroid without crossing the retina and (2) minimizing factors that could reduce control over MN placement in the tissue using an approach we previously developed to target injections into the SCS of the eye.^36^

**FIGURE 2:**
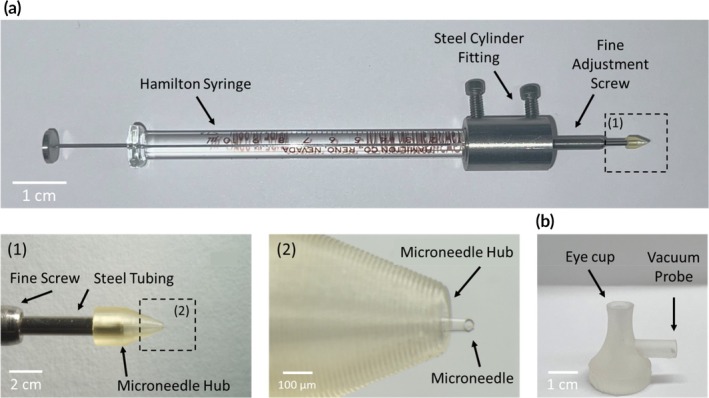
Subretinal delivery technique developed for injections in rodents and rabbits. (a) Microneedle (MN) injector featuring an ultra‐small MN and a needle hub designed for precise penetration across sclera and choroid; magnified view shows the MN and hub with greater resolution. The MN is housed in a 12‐mm long piece of steel tubing and connected to a 10‐μL Hamilton Syringe via a fine adjustment screw and a steel cylinder fitting. (b) Custom‐made 3D‐printed vacuum probe used for eye stabilization during injection.

We optimized MN length considering the anatomy of our four animal models: mouse, rat, guinea pig and rabbit, by accounting for the total thickness of conjunctiva, sclera and choroid that a MN needs to cross to reach the SRS (Table S1). This yielded optimal MN lengths of 120, 220, 300, and 1100 μm for the mouse, rat, guinea pig, and rabbit, respectively, which we precisely controlled by mounting the MN in an adjustable holder with a fine adjustment screw to control the exposed MN length (Figure 2a). We also used a tip OD of 110 μm (with an inner diameter [ID] of 100 μm) and bevel angle of 55° to facilitate localizing the injection in the SRS, based on our prior findings that these parameters optimized SCS injection by balancing effective tissue penetration and injection localization.^36^ In these studies, we used glass micropipettes to fabricate MNs because of their ease of tunability and optimization of needle geometry. Notably, the tip OD of our MNs (~0.1 mm OD) was comparable to that of 41G needles commonly used in retinal surgeries.^37^

Although this study did not focus on delivering therapeutic agents like stem cells or viral vectors for gene therapy, we believe our MN design (with a tip ID of 100 μm) can accommodate drug, particle and cell payload applications. Previous studies have successfully used similarly sized ultrafine needle tips (e.g., 35–41 G needles) for subretinal stem cell or viral vector injections.^9^, ^38^, ^39^, ^40^ Moreover, shear stresses that are usually the cause of damage to cells and biomolecules during injection are expected to be small in these MNs. Because MN length is so short, prior modeling has shown that fluid flow is undeveloped, meaning that the velocity profile is relatively flat.^41^ It is the gradients in velocity associated with fully developed flow having a characteristic parabolic velocity profile that cause shear stress, which are not present in undeveloped flow in MNs.

We next maximized control over MN placement in the tissue during MN insertion and injection in two ways. First, we designed the hub from which the MN protruded with a tapered geometry to facilitate perpendicular insertion by providing better visualization of the MN contacting the ocular surface (Figure 2a). This minimized oblique insertions that could alter the MN penetration pathway across the tissue. Upon contact with the ocular surface, the hub also restricted scleral deformation to a highly localized area immediately surrounding the injection site, thus preventing deformations across large areas that otherwise lead to poor MN penetration. Second, we used a 3D‐printed eye cup holder connected to a mild vacuum to stabilize the eye from any movement during injection that could similarly interfere with complete and controlled MN scleral penetration (Figure 2b).

We used this optimized injection technique for subretinal delivery in mice, rats, guinea pigs and rabbits, and assessed successful subretinal delivery based on formation of a fluid bleb in the SRS^2^ (Figure S1). We attempted 65 subretinal injections in rats and achieved bleb formation 85% of the time on the first attempt (55/65), indicating the reliability of subretinal injection. For perspective, while the specific count of eyes that are excluded due to injection failure is often not reported, the rate of unsuccessful injections by conventional methods can reach up to 50% in rodents because of the intricate nature of traditional subretinal injections.^42^, ^43^, ^44^ In addition, clinical experience shows that repeated attempts to achieve subretinal injection are common, even in the hands of highly experienced retinal surgeons guided by surgical microscopes due to the complexity of the conventional transvitreal injection method.^45^

**FIGURE 3:**
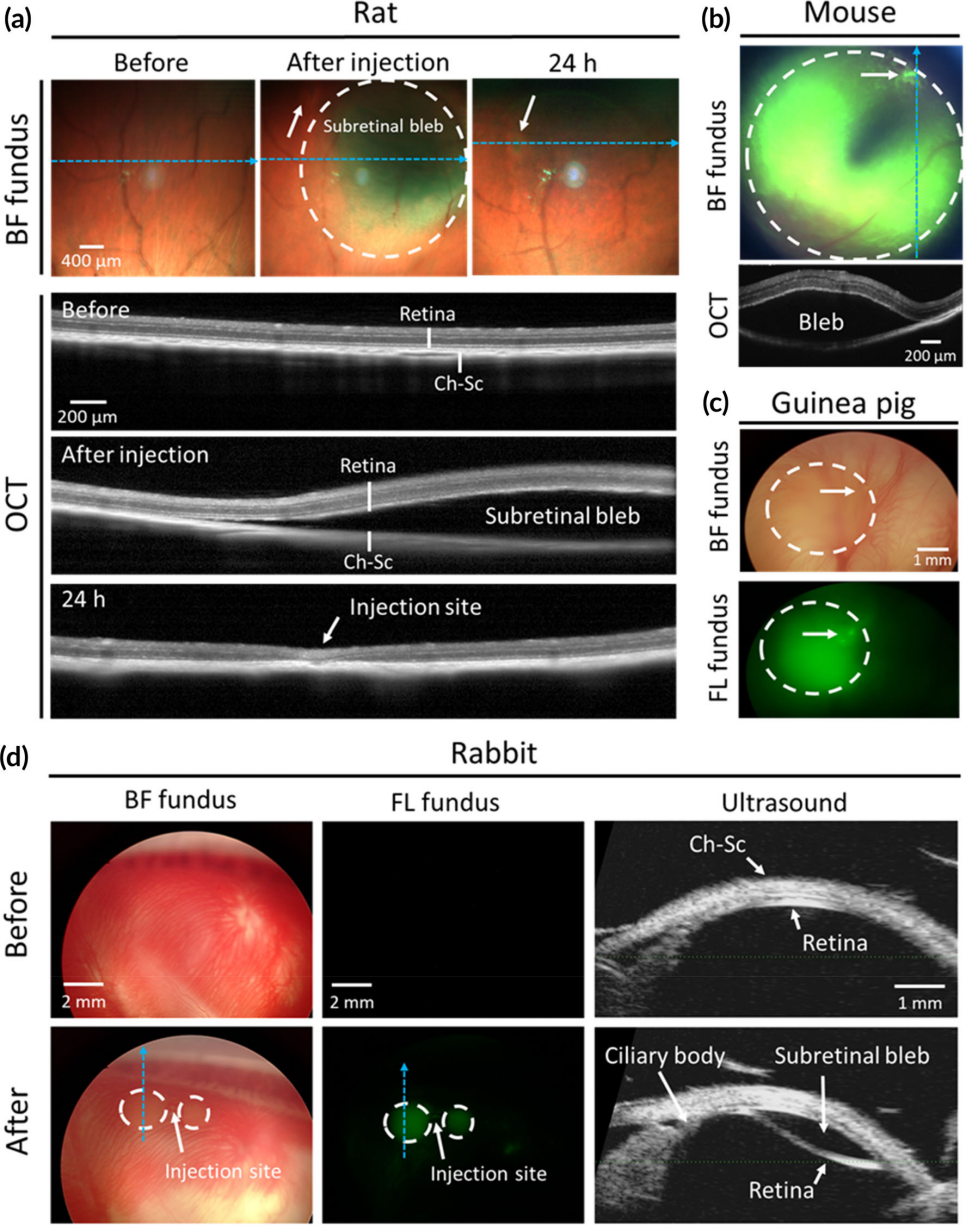
Transscleral subretinal injection using microneedles in rodents and rabbit in vivo. (a) Representative fundus and OCT images taken before, immediately after, and 24 h after injection of 2 μL Hank's balanced salt solution (HBSS) in rat. (b) Representative fundus and OCT images taken immediately after injection of 1 μL HBSS containing 0.025% (w/v) 200 nm green‐fluorescent nanoparticles in mouse (*n* = 7). (c) Representative brightfield and fluorescent fundus images taken immediately after injection of 8 μL HBSS containing 0.025% (w/v) 200 nm green‐fluorescent nanoparticles in guinea pigs (*n* = 4). (d) Representative fundus and ultrasound images taken before and immediately after injection of 100 μL HBSS solution containing 0.05% (w/v) 200 nm green‐fluorescent nanoparticles in rabbits (*n* = 3). OCT and Ultrasound images approximately correspond to the dashed blue arrow in fundus images. Dashed circles show the area of subretinal bleb. White arrows indicate the injection site. OCT images correspond to the dashed blue line in fundus images. BF, brightfield; Ch‐Sc: choroid‐sclera; FL, fluorescent; OCT, optical coherence tomography. The complete study included *n* = 65 rat eyes, *n* = 7 mouse eyes, *n* = 4 guinea pig eyes and *n* = 3 rabbit eyes.

Using our subretinal injection method, bleb formation was readily evident by brightfield fundus imaging (Figure 3a). Optical coherence tomography (OCT) imaging further confirmed bleb formation that was localized at the SRS (Figure 3a). In addition to rats, we conducted proof‐of‐concept studies in different animal species, successfully achieving subretinal delivery in seven mice (seven injections, unilateral) (Figure 3b), two guinea pigs (four injections, bilateral) (Figure 3c), and two rabbits (three injections, bilateral) (Figure 3d). This illustrates the versatility of our technique across various animal species, including deep‐orbited rabbits. These injections in mice, guinea pigs and rabbits were limited to proof‐of‐concept demonstrations without evaluating injection success rate or optimizing injection volume or bleb size. For perspective, prior studies indicate that the tiny size of rodent eyes makes subretinal injection much harder than in larger human eyes.^42^, ^43^, ^44^, ^46^ In this study, we also found that subretinal injections in rabbit eyes were more easily executed due to their larger eye size and robust structure that provided better visualization and control of MN insertion into sclera compared to rodent eyes. It is worth noting that in some cases (e.g., Figure 3d), the subretinal bleb did not co‐localize with the injection site. Especially when performed close to the limbus, injections often produced two blebs extending laterally from the injection site. This phenomenon may be influenced by anatomical factors affecting fluid distribution in the SRS, although this was not specifically investigated in our studies.

Consistent with subretinal injection by other methods,^9^, ^16^, ^27^ the induced subretinal bleb was transient and self‐resolved within 24 h post‐injection, based on fundus and OCT imaging (Figure 3a). Delivery was targeted to the SRS without intravitreal leakage or SCS delivery, as confirmed by OCT and fundoscopy examination (Figure 3). The injection process took <1 min to perform (i.e., including MN insertion, fluid injection and MN removal) and did not require an operating microscope (i.e., it was performed by visual observation without magnification), vitrectomy or multiple ocular punctures.

### 2.3 Acute and long‐term safety examinations after subretinal injection by microneedle

To better understand the safety of subretinal injection by MN, we conducted a detailed histological analysis at several timepoints between 24 h and 6 weeks post‐injection to identify both acute and long‐term outcomes. In this study, we used histology to assess retinal and choroidal tissue microanatomy by hematoxylin and eosin staining, and specifically examined RPE and photoreceptor outer segment cells by RPE65 and RHO antibody staining, respectively. We also looked for macrophage/microglial cells, neovascularization, and apoptosis by immunohistochemical staining with IBA1 antibody, GS Lectin, and TUNEL. The integrity of RPE structure was examined by confocal microscopy of RPE flatmounts, and retinal function was assessed by electroretinogram (ERG).

**FIGURE 4:**
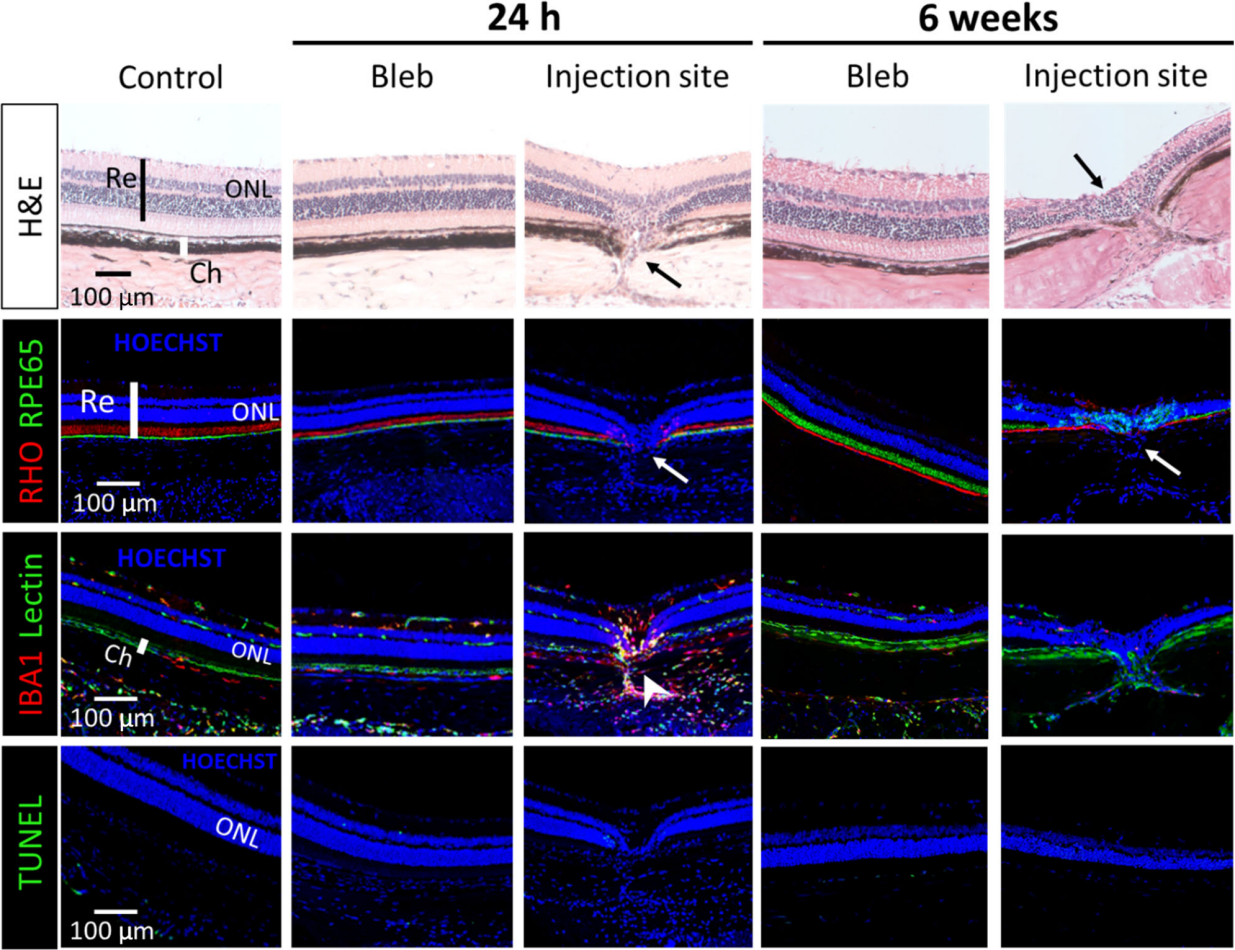
Acute and long‐term safety analysis after subretinal delivery of Hank's balanced salt solution via trans‐scleral microneedle injection in rats in vivo. Hematoxylin and eosin (H&E) staining revealed evidence of penetration across choroid and retinal pigment epithelium (RPE) that was highly localized at the microneedle (MN) puncture site and did not expand over time. Immunohistochemistry (IHC) staining with RPE65 and RHO antibodies showed focal loss of RPE and photoreceptor outer segment, respectively, at the injection site that did not expand over time. IHC staining with IBA1 indicated mild macrophage/microglial cell reaction at 24 h only at the puncture site that disappeared within 10 days (Figure S2). No evidence of neovascularization or apoptotic cell death was found, as shown by GS Lectin and TUNEL staining, respectively. Nothing notable was observed in the bleb regions. The study was conducted in *n* = 17 rat eyes including *n* = 3 eyes examined at 24 h, *n* = 6 eyes at 6 weeks, *n* = 4 at 72 h (Figure S2) and *n* = 4 at 10 days (Figure S2) timepoint. Contralateral eyes were used as control. Ch, choroid; ONL, outer nuclear layer; Re, retina.

Histological examinations revealed nothing notable over the course of 6 weeks anywhere in the bleb region other than at the site of MN puncture. At that location, microscopic evidence of penetration across choroid and RPE was evident, but no puncture across retina was seen. This micron‐scale puncture did not expand over time (Figure 4, Figure S2). Electron microscopy imaging at 6 weeks further showed highly localized MN puncture and normal tissue morphology immediately next to the injection site (Figure S3). Mild macrophage reaction was seen just at the site of puncture at 24 h but disappeared within 10 days. There was no evidence of neovascularization or apoptosis (Figure 4, Figure S2).

Additionally, to account for the effect of injection on the retina, we counted the number of cell nuclei in the outer nuclear layer (ONL) and found limited photoreceptor cell loss localized to the MN puncture site of some eyes with no notable loss anywhere else in the bleb region (Figure S4). This loss of a few ONL and photoreceptor cells at the injection site may have been caused by the fluid force coming out of the needle tip that may have penetrated retinal layers. The injection flow rate in our studies was approximately 0.3 μL/s, manually controlled without the use of an infusion pump. A slower injection rate might help mitigate this effect, although this was not tested in our study. For context, canine and non‐human primate studies on transvitreal subretinal injections have reported similar results, including limited ONL thinning and RPE abnormalities near or at the retinotomy site, which are attributed to the surgical procedure.^13^, ^47^, ^48^, ^49^ In rare cases, evidence of injection into the inner retinal layers was seen near the injection site that was associated with localized histological changes to retinal tissue (Figure S5). This contrasts with convention transvitreal injection, which punctures a hole across the retina every time.

**FIGURE 5:**
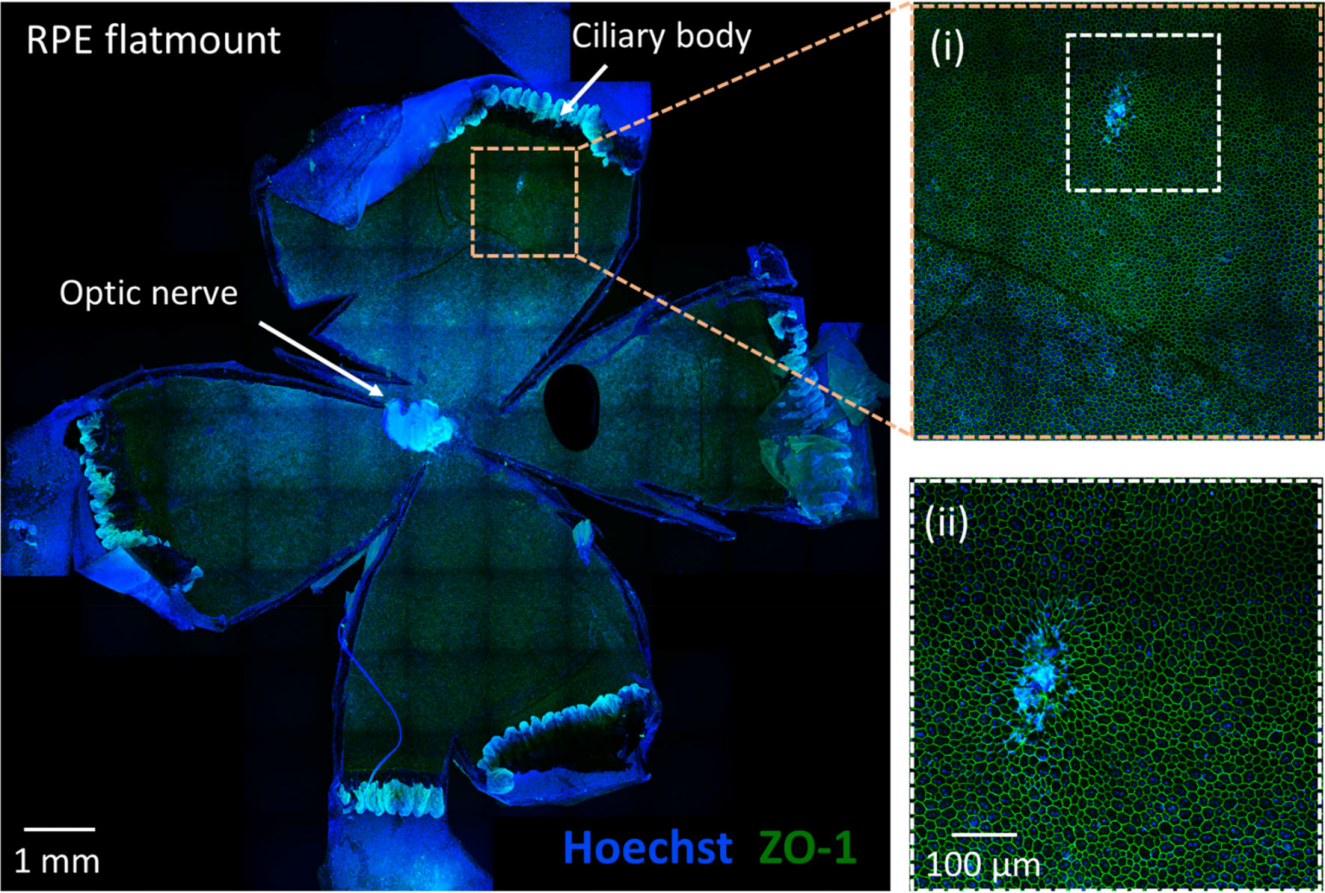
Representative confocal microscopy image of a retinal pigment epithelium (RPE) flatmount 6 weeks after subretinal delivery of Hank's balanced salt solution via trans‐scleral microneedle (MN) injection in rats in vivo. Flatmount exhibits staining of ZO‐1 (green), a tight junction protein of RPE cell membrane, and cell nuclei (Hoechst, blue). Insets (i, ii) provide progressively magnified views of the MN puncture site. The study included *n* = 4 injected eyes and *n* = 3 contralateral eyes as control. Morphometrical analysis of RPE flatmounts images is shown in Figure S6.

Ocular tissue flatmounts 6 weeks after subretinal delivery provided a holistic view of injection effect on the fundus and RPE monolayer cell sheet. The MN puncture site could be seen in a tiny area in the peripheral fundus, consistent with our cross‐sectional histological observations (Figure 5). To extract quantitative data, flatmount images underwent cell segmentation image analysis to identify individual RPE cells. In doing so, we found cell loss limited to the puncture site area averaged 0.027 ± 0.012 mm^2^. This area correlated to a nominal diameter of 186 ± 43 μm (assuming a circle) which was similar to the size of the MN tip used in the injections (110 μm in OD), and it corresponded to a loss of 36 ± 17 RPE cells. No morphometric alterations were found anywhere else in the bleb region (Figure S6).

### 2.4 Characterization of ocular bleeding after subretinal injection by microneedle

During MN device development above, we characterized ocular bleeding due to needle insertion. Here, we further characterized this bleeding after the complete process of subretinal injection. We quantified possible bleeding in 31 rat eyes by performing serial cross‐sectional OCT images across the bleb area (Figure S7). There was no evidence of intraocular or extraocular hemorrhage in any eyes (0/31). In 45% of cases (14/31), there was no evidence of any blood spot at all, and in the remaining cases a highly localized and microscopic blood spot was seen at the site of MN puncture (Figure S7B). The microscopic blood spots were found to have an average blood volume of 22 ± 38 nL, and they spontaneously self‐resolved within a few days in all cases (Figure S8).

In another experiment, five consecutive trans‐scleral subretinal injections in the same eye produced no apparent intraocular bleeding, further validating the low incidence of choroidal bleeding (Figure S9). In mice, guinea pigs, and rabbits, we did not observe any bleeding (i.e., neither hemorrhage nor blood spots) (14/14) upon visual inspection of fundus and OCT images taken immediately after injection.

We conclude that subretinal injection by MN produced no evidence of choroidal hemorrhage in rats, mice, guinea pigs and rabbits. In about half of cases, a tiny, transient blood spot was seen in rats, but was never seen in mice, guinea pigs or rabbits. It should be noted that although the conventional (i.e., transvitreal) subretinal injection does not require penetrating the choroid, there is often subretinal or vitreous bleeding due to accidental damage to retinal blood vessels or penetration into the choroid at the retinotomy site.^8^, ^13^, ^20^ Multiple studies conducted on canine and non‐human primate models have documented instances of localized bleeding near the retinotomy site, which typically resolves on its own.^47^, ^48^, ^50^, ^51^ Prior studies in humans report that localized, thin‐layered subretinal bleeding is typically benign with no apparent impact on visual acuity in regions outside the fovea.^33^, ^34^

### 2.5 Repeated subretinal delivery by trans‐scleral microneedle injection

**FIGURE 6:**
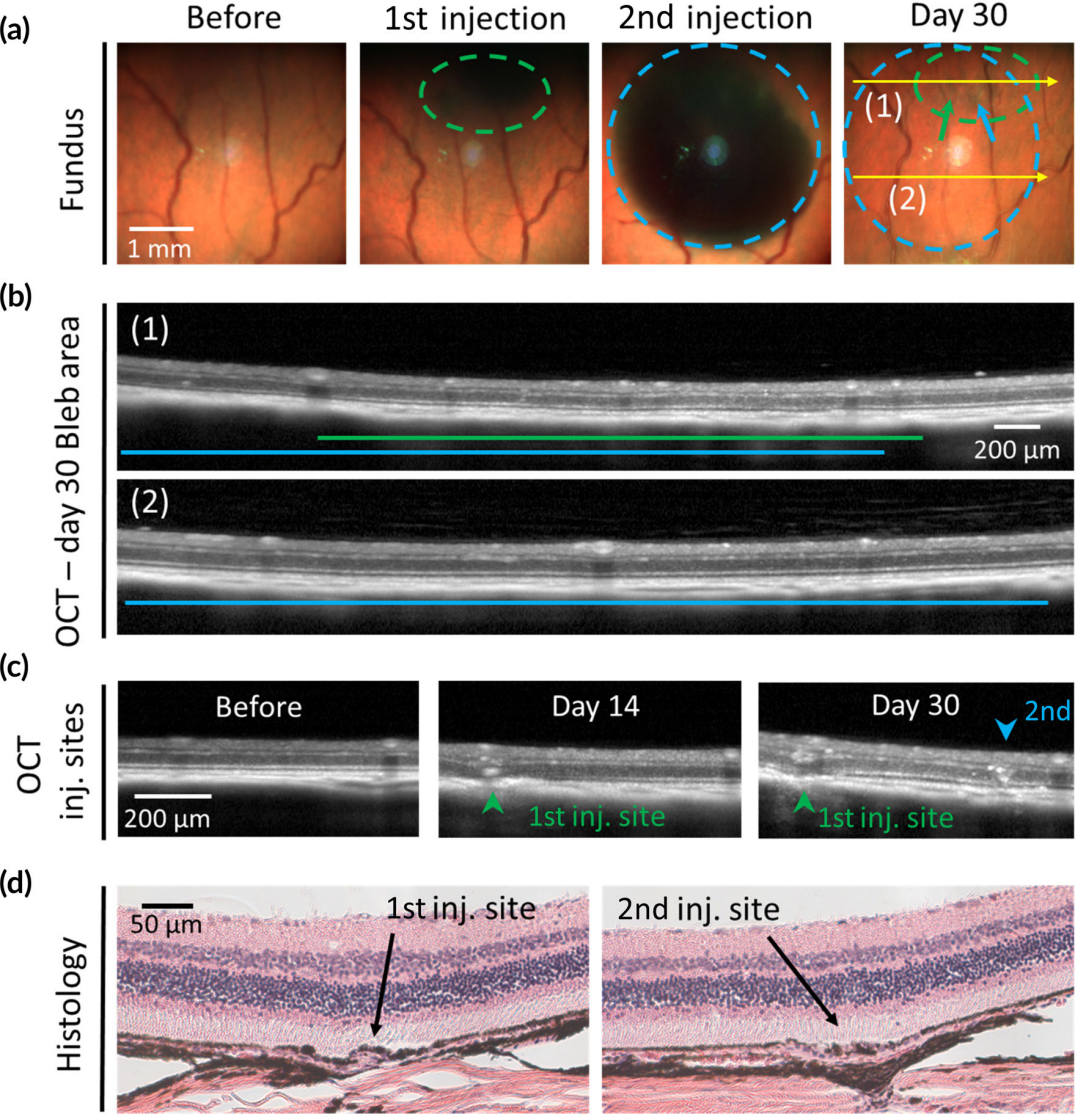
Sequential subretinal delivery of Hank's balanced salt solution (HBSS) via trans‐scleral microneedle injection in rats in vivo. (a) Representative brightfield fundus images of an eye that received two subretinal injections, 10 days apart, where the second bleb (dashed blue circle) overlapped and expanded the area covered by the bleb from the first injection (dashed green circle). Green and blue arrows in the fundus image on day 30 indicate the first and second microneedle puncture sites, respectively. (b) Representative optical coherence tomography (OCT) images of the bleb areas taken on day 30 show ocular sections corresponding to a region of overlapped spreads (1, dashed yellow line in [a]) or a single spread (2, dashed yellow line in [a]). Green and blue lines indicate the area of spread by first and second injections, respectively. (c) Representative OCT images showing ocular tissue structure at the injection sites. Microneedle puncture was highly localized, did not penetrate across retina and did not grow over time. Retinal structure between the injection sites appeared normal despite the close proximity of the two injections. Retina maintained its structural integrity despite being repeatedly detached at the first injection site. (d) Representative histological tissue sections obtained upon termination of the study on day 30 highlighting the two microneedle puncture sites with highly localized penetration across choroid and RPE and no apparent retinal disruption. No notable abnormalities were observed in the bleb regions. The eyes received subretinal administration of 1 and 3 μL HBSS solution in the first and second injections, respectively. The study included *n* = 10 eyes. Additional images and analysis of sequential subretinal injections are shown in Figure S10.

Repeated subretinal administration may be needed to boost a treatment's effect over time, or to expand the therapeutic effect to additional areas not targeted in prior injection.^4^ Driven by the simplicity and tolerability of the injection procedure shown in the above studies, we hypothesized that trans‐scleral MN injection may enable sequential subretinal delivery in the same eye. To assess this hypothesis, we performed a second subretinal injection in the same eye 10–14 days after the first injection. In some cases, both injections were done near each other to increase the likelihood of overlapping blebs resembling a booster scenario (Figure 6). In other cases, injections were made far apart so that the second bleb expanded the retinal coverage (Figure S10). Thirty days after the first injection (~2 weeks after the second injection), both blebs had resolved, and we found no abnormality in the areas of two overlapping spreads or individual bleb formation. Consistent with our previous findings, evidence of highly localized MN puncture without retinal perforation could be seen at both injection sites, which did not expand to the neighboring areas over the course of study (Figure 6, Figure S10). Notably, even when the two MN puncture sites were extremely close (~300 μm apart), there was no evidence of interactive effects, such that both puncture sites remained contained with no retinal abnormalities in between. It is worth highlighting that in this case, the second injection had raised a bleb detaching the retina at the first puncture site. This repeated detachment had no synergistic negative effect, and retinal structure at the first puncture site appeared undisturbed (Figure 6).

### 2.6 ERG examinations after subretinal injection by microneedle

**FIGURE 7:**
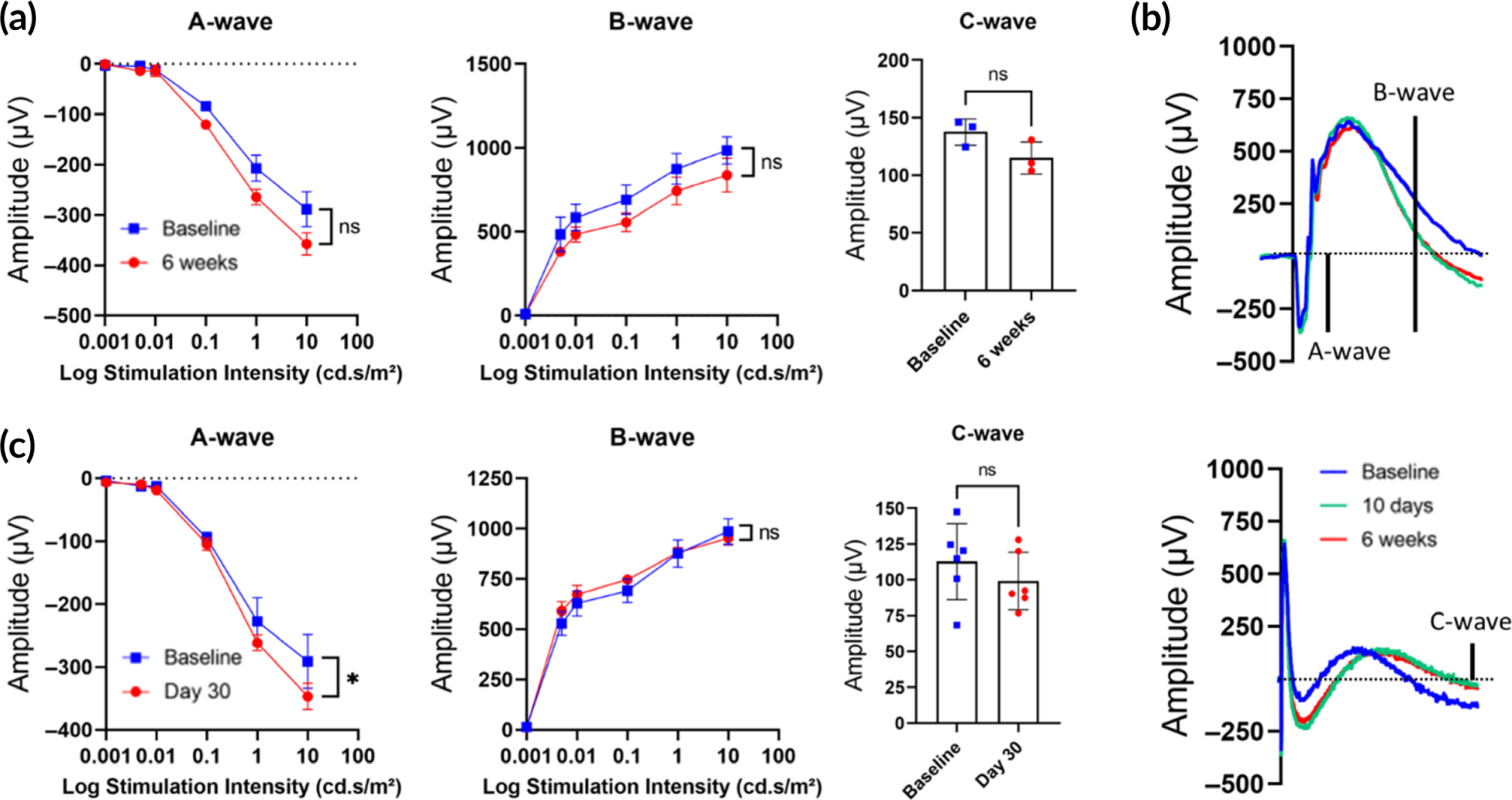
Electroretinogram (ERG) responses after subretinal delivery of Hank's balanced salt solution via trans‐scleral microneedle (MN) injection in rats in vivo. (a) ERG responses collected 6 weeks after a single subretinal injection showing no reduction in amplitude of ERG waves (*n* = 3 eyes). (b) Representative complete ERG waveform for an eye with the highest choroidal bleeding (118 nL) after a single subretinal injection indicating no reduction in ERG amplitude up to 6 weeks (*n* = 3 eyes). (c) ERG responses obtained from eyes that received two sequential subretinal injections showing no change in ERG response at day 30 compared to baseline (*n* = 6 eyes). A‐wave, B‐wave and C‐wave indicate photoreceptor cell, bipolar cell and retinal pigment epithelium cell responses, respectively. ERG data at 24 h, 72 h and 10 days after subretinal injection are shown in Figure S11. Differences between the ERG amplitudes were assessed using a paired *t*‐test (only the highest amplitudes were compared).

We collected full‐field ERG responses of the retina as a measure of retinal functionality.^47^, ^48^, ^49^, ^52^ Data obtained at various timepoints following a trans‐scleral subretinal injection by MN revealed no reduction in ERG waves over the course of the 6‐week study (Figure 7a, Figure S11) even when choroidal bleeding occurred (Figure 7b). Sequential subretinal injections also did not compromise the ERG response (Figure 7c).

## 3 DISCUSSION

### 3.1 Unmet need for an improved subretinal delivery

The method of drug administration is a defining factor in the success of eye therapies and patient outcomes,^9^ which motivated this study to improve subretinal delivery. Invasive delivery methods can damage sensitive ocular tissues and severely compromise treatment outcomes of an otherwise efficacious therapeutic agent. This is the case for many emerging gene and stem cell‐based ocular therapies that require subretinal injection, a complex and invasive surgical procedure that is needed to target retina and RPE.^2^, ^17^

The first Food and Drug Administration‐approved gene therapy product, for instance, is an ocular treatment administered by subretinal injection of a viral vector. It can successfully restore vision in patients with a hereditary blinding disorder but was associated with serious adverse effects such as cataracts, retinal tears and macular holes that were mainly attributed to the surgical procedure not the viral vector itself.^4^, ^53^

Attempts to replace subretinal injection with a safer and simpler delivery technique have had limited success. Studies have assessed intravitreal and SCS injections, both of which are office procedures that do not require vitrectomy and do not involve contact with neural retina, which means that they are not targeted and, as a result, have limited retinal bioavailability.^11^, ^14^

### 3.2 Development of a minimally invasive trans‐scleral microneedle injector

In the present work, we propose an injection method to perform subretinal delivery via a trans‐scleral MN in a simple, safe, and reliable manner. Our approach relies on two key capabilities: first, precise placement of the MN tip in the SRS, and second, minimized disruption of ocular tissue, especially choroidal blood vessels and neural retina. To achieve these outcomes, the following features were incorporated into our injection technique: (1) a MN whose length was tightly controlled to match the thickness of sclera and choroid, (2) a 3D‐printed vacuum probe to stabilize the eye, and (3) perpendicular MN insertion into the eye. These elements together achieved precise needle placement in the following way.

The controlled MN length enabled penetration across sclera and choroid into the SRS but physically inhibited deeper penetration into neural retina. The vacuum probe prevented any eye movement during injection that could lead to poorly controlled MN penetration. It was then critical for the MN to penetrate tissue perpendicularly because otherwise the needle would have to traverse a longer (diagonal) distance to reach the SRS if inserted at oblique angles.

In addition, the combined effect of these features also minimized disruption of ocular tissue. The ultra‐small size of MNs, measuring 110 μm in diameter at the tip, enabled microscopic puncture across tissue layers. Eye stabilization lowered collateral damage to surrounding tissues beyond the puncture site by localizing the MN to its intended location. Lastly, perpendicular insertion provided the shortest path to reach the SRS, thus minimizing needle track size in tissue.

### 3.3 Targeted subretinal delivery

Injections using the proposed technique achieved targeted subretinal delivery in three rodent species, i.e., mice, rats, and guinea pigs, as well as in rabbits, as confirmed by subretinal bleb formation near the injection site.^2^ The associated retinal detachment was transient and self‐resolved within 24 h post‐injection. We found no evidence of retinal perforation or vitreous leakage indicating the MN's effectiveness in controlling tissue penetration. The injection procedure was straightforward, took about 1 min to perform per eye without needing any special equipment such as an operating microscope.

Despite possible variations of scleral and choroidal thickness between animals, we found that a single MN length achieved subretinal delivery in all animals of each species. We think two factors may have mitigated the effect of tissue thickness variation: (1) we consistently performed the injection at the same location, i.e., 1–2 mm (rodents) and 5 mm (rabbits) posterior to the limbus in the superior hemisphere and (2) animals were roughly of the same age when receiving the injection. We realize, however, that these uniform conditions may not be applicable to humans because many factors besides age and location can influence ocular tissue thickness. To address this, the MN length could be personalized by measuring scleral and choroidal thickness at the intended injection site by OCT or ultrasound imaging prior to the injection to ensure precise subretinal delivery without over‐ or under‐penetration.

### 3.4 Safety examinations

Detailed examination of ocular tissue morphology revealed no notable adverse effect in the bleb regions other than evidence of a highly localized MN puncture site in the retinal periphery that did not grow over time. At this location, microscopic RPE cell loss correlating to an average of 36 cells was observed, which seems unlikely to affect visual acuity, especially in the peripheral retina. Localized ONL disorganization and limited loss of photoreceptors were sometimes found at this location too. No neovascularization or apoptotic cells were found, and injections triggered only a mild, transient macrophage/microglia response that resolved within 10 days. These microscopic changes did not have noticeable effect on retinal functionality as assessed by ERG.

In those occasional cases where we noted retinal changes characterized by disorganization of ONL and focal loss of photoreceptor outer segment in the vicinity of the injection site, we found evidence of intraretinal delivery that expanded the inner retinal layers and may have caused the observed damage (Figure S5). In those cases, intraretinal delivery is unlikely to have been caused by deeper penetration on MNs into inner retinal layers because the fixed length of the MNs physically prevents such an outcome. We suspect that the fluid jet stream exiting the MN orifice may be responsible for perforation into retinal layers and partial intraretinal delivery in those cases. Slower, less‐forceful injections could ameliorate that problem.

### 3.5 Other safety examinations

Contrary to the general paradigm that any penetration involving choroidal vasculature risks causing hemorrhage,^30^ we hypothesized that choroidal bleeding is proportional to the extent of damage to its vasculature upon penetration, such that sufficiently small needles could avoid hemorrhage. Indeed, our results showed no incidence of choroidal hemorrhage after subretinal MN injection. Self‐contained and localized blood spots were sometimes seen in rats as a thin layer of RBCs spanning a few hundred microns in the SRS near the injection site in the retinal periphery, which resolved by 6 weeks with no apparent detrimental effect on retinal morphology other than rare cases of sparse, microscopic subretinal pigment abnormalities. This aligns with literature reports that thin‐layered localized subretinal bleeding does not affect visual acuity in humans especially when outside the central macular region.^33^, ^34^

This minimally invasive outcome was likely achieved by the ultra‐small size of MNs that reached the SRS by a microscopic puncture through the choroid vasculature, thereby minimizing the chance of bleeding. In contrast, high incidence of choroidal hemorrhage following trans‐choroidal insertion of hypodermic needles with two–four times bigger tip diameter provided further evidence supporting the above hypothesis. However, factors beyond the larger needle diameter, such as the needle material, wall smoothness or other characteristics, may have also contributed to the higher incidence of choroidal hemorrhage. Furthermore, perpendicular insertion and eye stabilization may have also contributed to avoidance of hemorrhage, although these effects were not separately tested in this study.

We note that 45% of subretinal injections caused no detectable bleeding at all, while other injections had microscopic blood spots despite having been performed using identical MNs. We speculate that the size of choroidal vasculature is a confounding factor in determining the likelihood of bleeding that could explain the discrepancy in blood spot incidence observed here. Choroid consists of an intertwined network of blood vessels of various sizes.^54^ Regardless of needle size, penetrating choroid at a site with small blood vessels in the choriocapillaris would less likely cause a blood spot compared to penetrating a site where a larger vessel is nicked. We suspect that subretinal injections that caused blood spots in this study may have encountered a larger choroidal vessel compared to injections with no bleeding at all.

It is notable that we achieved so little bleeding simply by blind MN insertion with no visualization of the underlying choroidal vasculature, further highlighting the effectiveness of the ultra‐small MNs. Additionally, this finding offers a strategy to reduce this risk even further by identifying an optimal puncture site ideally devoid of medium and large blood vessels prior to the injection using non‐invasive imaging methods such as OCT or ultrasound.

### 3.6 Study limitations

This study has a number of limitations. First, it was conducted in a moderate number of rats (*n* = 45 for both injection and safety studies) and a small number of mice (*n* = 7), guinea pigs (*n* = 2), and rabbits (*n* = 2). Additional studies are needed using larger numbers of animals, in additional species (including humans), and by other investigators to assess the broad utility of trans‐scleral MN delivery to the SRS. While the safety examination was detailed—and went well beyond the level of safety analysis in most prior studies of subretinal injection^9^, ^18^, ^42^, ^47^, ^48^, ^49^—more study is needed (e.g., intraocular pressure assessment following SRS injection), especially over longer times, after more than two repeated injections, and in animal models more closely resembling deep‐orbited humans, as well as human themselves.

In this study, eyes were proptosed before injection to expose the injection area on the small animal eyes. Future research should explore extending our subretinal injection method to deep‐orbited eyes for posterior delivery without proptosis. We speculate that the larger size of human eyes compared to rodents will allow greater scleral access, and simple maneuvers like rotating the eyeball may enable access up to the equator,^18^ eliminating the need for proptosis. While injecting near the macula may require extensive surgical manipulations or novel MN device designs, biomechanical techniques, such as optimizing fluid injection parameters, could direct fluid flow in the SRS to facilitate macular delivery from an injection site at the equator or a more anterior location, like we have done previously for MN injections in the SCS.^55^

This study did not include comparison to a conventional subretinal injection because experience with transvitreal injections is well documented in the literature and outcomes from transvitreal injections are highly dependent on technique, especially in small animals.^42^, ^43^, ^44^ We attempted multiple transvitreal subretinal injections ourselves in rats, but all of them caused hemorrhage and did not form a bleb. We did not feel that these results would be suitable as a control, since others in the literature have made subretinal injections successfully, which further highlights the highly variable outcomes of conventional subretinal injection and the need for specialized training.

The focus of this study was on the safety and reliability of subretinal injection by our novel MN method. It did not, however, address delivery of therapeutic agents of interest, such as viral vectors for gene therapies or cells for stem cell therapies. The literature contains many reports of successful outcomes from such agents being delivered into the SRS, suggesting that our subretinal delivery method should enable the same successful outcomes, but that remains to be shown in future studies. Although our MNs are small, their 100 μm ID is large enough to allow passage of proteins, virus particles and cells.

To address the issue of cell passage through MNs, we flowed cultured human RPE cells (ARPE‐19) through our optimized MNs for SRS injection and found that this passage did not affect cell viability compared to untreated cells, as assessed by histochemical (trypan blue) staining (data not shown).

We also performed an initial assessment of the feasibility of trans‐scleral MN injection to deliver adeno‐associated virus (AAV) vectors to the SRS for potential gene therapy applications, we performed subretinal injection into rat eyes with AAV2 encoding green fluorescent protein (GFP). In vivo fluorescent retinal fundoscopy along with histological staining at day 30 post injection revealed successful expression of GFP at the injected area in RPE and photoreceptor cell nuclei (i.e., ONL), without evidence of retinal abnormalities (Figure S12). We noted that strong GFP signal was detected in some cells, but overall expression appeared patchy, which may be attributed to the low viral concentration used in this study (~1.8 × 10^10^ viral genomes per eye).

These results provide proof‐of‐concept evidence of trans‐scleral MN delivery of a viral vector into the SRS to cause subretinal transduction leading to expression of a reporter protein. However, additional studies and further optimization will be needed to better assess the use of this subretinal delivery method to administer therapeutic agents and achieve biological outcomes.

### 3.7 Translational considerations

Subretinal injection by MN was identically performed in mice, rats, guinea pigs and rabbits, with the only change being adjusting MN length to accommodate species‐specific tissue thicknesses, despite the differences in size and anatomy of the eye in those species. We found that the injections were more straightforward to perform in larger eyes, perhaps because injections in smaller, more‐fragile eyes demand a higher degree of precision and finesse with lower tolerance for variation. We hypothesize that studying and optimizing the injection method in the more‐challenging rodent eyes may enable the critical level of precision needed to reliably perform subretinal delivery in larger eyes, such as humans. Our detailed, microscopic safety examinations in rats revealed an excellent safety profile with microscopic, self‐resolving, and highly localized disruption of ocular tissues seen only at the site of injection. Given that procedure‐related ocular injuries are more likely to occur in smaller and fragile rodent eyes,^4^ the proposed method may have an even more favorable safety profile in human eyes, which are structurally more robust. Albeit limited to imaging assessments over the course of only 6 weeks after injection, these results show the ability of the proposed technique to overcome the two most daunting challenges of trans‐scleral subretinal delivery: choroidal hemorrhage and retinal perforation. These encouraging findings demonstrate the versatility and scalability of transscleral MN injection for use not only in various animal species but also potential use in humans in the future.

## 4 CONCLUSION

This study developed a minimally invasive and non‐surgical method of trans‐scleral subretinal delivery by controlling precise MN penetration into the SRS and minimizing damage to choroidal vasculature. Injection in mice, rats, guinea pigs and rabbits reliably accomplished subretinal delivery without retinal perforation or choroidal hemorrhage via a simple, one‐step procedure that took about 1 min per injection and required only a single, shallow eye puncture with no surgical microscope or vitrectomy. The presented technique equips ophthalmology researchers with a tool that can be used across multiple species to expedite preclinical development of novel subretinal therapies. More importantly, this work lays the foundation for the development of a robust and minimally invasive method of subretinal injection in humans that might be routinely performed possibly as an office procedure.

## 5 MATERIALS AND METHODS

### 5.1 Subretinal injector design and fabrication

To fabricate hollow MNs, fire‐polished aluminosilicate glass pipettes (OD 1 mm, Sutter Instrument, Novato, CA, USA) were pulled using a micropipette puller (P‐97, Sutter Instrument). The resulting MNs were beveled at 55° to a tip orifice size of 110 μm utilizing a beveler device (BV‐10, Sutter Instrument). Aluminosilicate glass pipettes were selected for MN fabrication due to their chemical inertness and ability to provide sufficient mechanical strength when pulled. Across the range of lengths tested in this study, the MN demonstrated excellent durability, with no instances of breakage observed. Other materials such as borosilicate glass pipettes may also be used for fabricating MNs but were not tested in this study.

Ethanol was then flushed through the MNs followed by two flushes of deionized water to clear the lumen from glass debris. Finally, MNs were individually housed in a 12 mm‐long piece of stainless‐steel tubing (OD 1.47 mm, wall thickness 0.2 mm, McMaster‐Carr, Douglasville, GA, USA) and connected to a 10 μL Hamilton syringe (#7653–01, Hamilton, Reno, NV, USA) via a fine screw fitting (M3‐0.1, Base Lab Tools, Stroudsburg, PA, USA). The extremely small thread on the screw fitting enabled fine adjustment of MN length protruding from the tubing by moving the steel tubing forward and backward along the needle length.

The needle hub and vacuum eye stabilizer were designed via computer‐aided design (Solidworks, Waltham, MA USA) and fabricated using a 3D‐printer (SLA Form 2, Formlabs, Somerville, MA, USA). Because of their contact with ocular surfaces, these parts were printed with the highest resolution to provide a smooth surface finish, which was confirmed by inspection through a stereomicroscope (SZX16, Olympus, Tokyo, Japan).

### 5.2 Animal studies

All animal procedures were approved by the Georgia Institute of Technology and Emory University Institutional Animal Case and Use Committee (Protocol numbers A100080, A100086, PROTO201700013, and PROTO202000009), which complied with the ARVO Statement for the Use of Animals in Ophthalmic and Vision Research. Rats, guinea pigs, and rabbits were obtained from Charles River Laboratories (Wilmington, MA, USA), and C57BI/6J mice were ordered from the Jackson Laboratory (Bar Harbor, ME, USA). Mice, guinea pigs, and rabbit experiments included a mix of both male and female animals, while rat experiments were conducted in female animals. Future studies in rats should include male animals to investigate injection outcomes across sex groups. Contralateral eyes were used as controls.

#### 5.2.1 Trans‐scleral insertion of hypodermic needles and microneedles to assess choroidal bleeding

In this terminal procedure, the incidence of intraocular and extraocular bleeding was assessed in 24 rat eyes (Long Evans rats, female, 8–12 weeks old, *n* = 12) following trans‐scleral insertion of hypodermic needles and microneedles into the SRS. The hypodermic needles tested included 33G needles (OD: 210 μm), 30G needles (OD: 310 μm), and 27G needles (OD: 410 μm), and the MNs tested had an OD of 110 μm. Each needle size was tested in six eyes (*n* = 3 rats, bilateral) and each eye received up to four insertions in the superior side unless intraocular or extraocular hemorrhage occurred. MN insertions were performed using the device shown in Figure 2. For hypodermic needle insertions, the same device was adapted by removing the microneedle hub and using the fine adjustment screw to control the exposed needle length, ensuring it matched the length of the MN insertions. Brightfield fundus and ocular surface images were taken before and immediately after each needle insertion to assess the extent of choroidal bleeding. Animals were then immediately euthanized via CO_2_ asphyxiation. Data from this study are shown in Figure 1.

#### 5.2.2 Trans‐scleral subretinal injection

Long Evans rats (female, 8–12 weeks old, *n* = 22) received trans‐scleral subretinal injection of 2 μL Hank's balanced salt solution (HBSS) (Mediatech, Manassas, VA, USA) in one eye. Baseline ERGs were recorded at least 7 days prior to the injection. Baseline fundus and OCT images were collected on the same day prior to the injection. To assess the acute and long‐term safety of the procedure, animals were divided into four groups, each of which were assessed after 24 h (*n* = 3), 72 h (*n* = 4), 10 days (*n* = 4), or 6 weeks (*n* = 11) post injection by ERG, fundus imaging, OCT, histology, RPE flatmount, and transmission electron microscopy. Animals were then euthanized via CO_2_ asphyxiation and eyes were harvested for post‐mortem analyses. Eyes collected at the 6 weeks timepoint were either sectioned for histological analysis (*n* = 6), sectioned for transmission electron microscopy imaging (*n* = 1), or dissected for RPE flatmount examination (*n* = 4). Data from this study are shown in Figures 3, 4, 5 and 7, Figures S2–S6 and S11.

C57BL/6J (Jax) mice (either sex, 4–6 months old, *n* = 7) received trans‐scleral subretinal injection of 1 μL HBSS containing 0.025% (w/v) 200 nm green‐fluorescent latex nanoparticles (excitation: 505 nm; emission: 515 nm; FluoSpheres, ThermoFisher Scientific, Waltham, MA, USA) in one eye. Fundus and OCT imaging were performed before and immediately after injection to assess injection success. Fluorescent particles were added to the injection solution for better identification of bleb area in fundus imaging. No post‐mortem safety examinations were conducted in mice. Data from this study are shown in Figure 3.

Hartley guinea pigs (either sex, 6–9 months old, *n* = 2) received bilateral trans‐scleral subretinal injection of 8 μL HBSS containing 0.025% (w/v) fluorescein (AK‐fluor 10%, Akorn, Lake Forest, IL, USA). Eyes were imaged by brightfield and fluorescence fundoscopy before and immediately after injection to assess injection success. No post‐mortem safety examinations were conducted in guinea pigs. Data from this study are shown in Figure 3.

New Zealand White rabbits (either sex, 3.0–3.5 kg, *n* = 2) received bilateral trans‐scleral subretinal injection of 100 μL HBSS containing 0.05% (w/v) 200 nm green‐fluorescent latex nanoparticles (FluoSpheres). Eyes were imaged by brightfield and fluorescence fundoscopy, as well as ultrasound biomicroscopy, before and immediately after injection to assess injection success. No post‐mortem safety examinations were conducted in rabbits. Data from this study are shown in Figure 3.

#### 5.2.3 Trans‐scleral subretinal injection by microneedles to assess choroidal bleeding

Bleeding was measured in 31 rat eyes (Long Evans rats, female, 8–12 weeks old) that had received trans‐scleral subretinal injection. Serial OCT images of the full bleb area taken immediately after injection were used to obtain calculate blot spot size by multiplying the thickness of each image (120 μm) by the sum of cross‐sectional areas of SRS containing blood measured from individual OCT images. Blood was easily identifiable as a white reflective region within the subretinal bleb. Blebs without blood appeared clear (black). All measurements were made using ImageJ (NIH, Bethesda, MD, USA). Data from this study are shown in Figures S7 and S8.

In a separate terminal procedure, five consecutive trans‐scleral subretinal injections were performed in one eye of a Wistar rat (male, 6–9 months). Injections were done 5 min apart and fundus imaging was performed immediately after each injection to assess injection success and choroidal bleeding. Data from this study are shown in Figure S9.

#### 5.2.4 Sequential subretinal administration

Long Evans rats (female, 8–12 weeks old) received two trans‐scleral subretinal injections of 1–3 μL HBSS solution 10–14 days apart unilaterally (*n* = 4, i.e., eight injections in four eyes) or bilaterally (*n* = 3, i.e., 12 injections in six eyes). Fundus and OCT imaging were performed before the first injection, before and after the second injection, and on day 30. ERG was only performed in the bilateral injection group as a baseline ERG at least 1 week prior to the first injection and a post‐injection ERG on day 30, after which the animals were euthanized by CO_2_ asphyxiation and eyes were collected for post‐mortem histological analyses. Data from this study are shown in Figures 6 and 7, Figure S10.

#### 5.2.5 Subretinal AAV2 delivery

Purified recombinant AAV2 viral vectors containing a cytomegalovirus promoter and GFP transgene were provided by Emory University Viral Vector core (Atlanta, GA, USA). Long Evans rats (female, 8–12 weeks old, *n* = 4) were injected in both eyes with 1 μL of HBSS containing AAV2 vectors at a titer of 1.8 × 10^13^ vg/ml (1.8 × 10^10^ vg per eye). GFP expression was assessed via fluorescence fundoscopy and OCT imaging conducted before and on day 30 post‐injection, after which the animals were euthanized by CO_2_ asphyxiation and eyes were collected for post‐mortem histological analysis. Data from this study are shown in Figure S12.

#### 5.2.6 Cell viability after passing through MNs

Ten microliter solutions of ARPE‐19 cells suspended in PBS were passed through our optimized MN with ID of 100 μm and were subsequently loaded onto a hemocytometer. Cell viability was measured using 0.4% trypan blue staining under light microscope. In another method, cell viability was measured using Calcein and Ethidium homodimer using fluorescent microscopy. The results were then compared with the control group in which the cell suspension had not been passed through a MN.

### 5.3 Subretinal injection procedure

#### 5.3.1 Anesthesia

Rodents were given intraperitoneal injection of ketamine:xylazine (80 mg/kg:8 mg/kg for rats and guinea pigs, 100 mg/kg:10 mg/kg for mice) to induce general anesthesia. A drop of 0.5% tetracaine hydrochloride ophthalmic solution (Amici Pharmaceuticals, Melville, NY, USA) or 0.5% proparacaine hydrochloride ophthalmic solution (Akorn, Buffalo Grove, IL USA) was applied to each eye for topical anesthesia. To dilate the eye, one drop of 1% tropicamide ophthalmic solution (Henry Schein, Melville, NY, USA) was applied and allowed 5 min to take effect. In Long Evans rats, a drop of 2.5% phenylephrine ophthalmic solution (Bausch & Lomb, Tampa, FL USA) was then applied to fully dilate the eye. To prevent dehydration, ample amounts of eye lubricants (GenTeal Tears, Alcon, Fort Worth, TX, USA, or Refresh Tears 0.5% carboxymethylcellulose ophthalmic solution, Allergan, Irvine, CA USA) were placed on the eye.

In rabbits, animals were placed in an induction chamber (Model 90100, Bickford, Wales Center, NY, USA) with 5% isoflurane (Isothesia, Henry Schein) and a 400 mL/min oxygen flow rate to induce general anesthesia. Once induction was achieved, isoflurane was administered at a reduced concentration (2%–3%) through a nose cone to maintain anesthesia throughout the procedure. The eye was then proptosed and irrigated with sterile saline followed by a 5% povidone‐iodine solution (Betadine, Alcon, Fort Worth, TX, USA) as an antiseptic. Eye drops containing 0.5% tetracaine hydrochloride (Amici Pharmaceuticals) and 1% tropicamide (Henry Schein) were then applied to achieve topical anesthesia and dilation, respectively.

#### 5.3.2 Trans‐scleral microneedle injection

To expose the scleral surface, the eye was proptosed using the latex glove method.^36^ Briefly, a small square piece (1.5 × 1.5 cm) was cut from a latex glove with a slit in the center into which the eye was placed such that it supported the eye and kept it proptosed. A custom‐made 3D printed eye probe was then placed on the inferior cornea‐conjunctiva, through which a gentle vacuum (Vacuum pump AIRPO D2028B, Karlsson Robotics, Tequesta, FL USA) was applied to secure the eye during injection. The MN was perpendicularly inserted into the eye at a location 1–2 mm (rodents) and 5 mm (rabbits) posterior to the limbus on the superior side, and liquid formulations were injected slowly (~0.3 μL/s) by pushing the plunger. The MN was kept in place for 30 s after injection to prevent reflux, after which the MN and latex glove were removed, and the vacuum was turned off.

Prior to injection, the MN length was adjusted to 120 μm for mice, 220 μm for rats, 300 μm for guinea pig and 1100 μm for rabbit injections. Each injection took about 1 min, starting from the glove proptosis until the vacuum was turned off and the glove removed. To assess injection success, fundus, OCT or ultrasound images were collected immediately (within 1 min) after injection. After that, animals were either allowed to recover from anesthesia and returned to their cages when ambulatory, or in terminal procedures, rodents were euthanized via CO_2_ asphyxiation while a single intravenous injection of euthanasia solution (pentobarbital 390 mg/mL, Euthasol, Virbac, FortWorth, TX) was used for rabbits. Animals were monitored throughout the course of study for any procedure‐associated adverse effects.

### 5.4 In vivo ocular imaging

For rats, confocal laser scanning ophthalmoscope fundus imaging, confocal laser scanning ophthalmoscope fluorescence fundus imaging, and OCT imaging were performed on the injected eye using a Heidelberg Spectralis OCT (Heidelberg Engineering, Franklin, MA, USA) or Retcam II fundoscopy system (Clarity Medical Systems, Pleasanton, CA, USA). A series of OCT images was recorded whenever possible, 120 μm apart, to capture a full cross‐sectional view of the site of subretinal injection. For mice, injected eyes were imaged immediately after injection via a Micron IV spectral domain OCT system with a fundus camera (Phoenix Research Labs, Pleasanton, CA, USA). For guinea pigs and rabbits, brightfield and fluorescence fundus images were collected from injected eyes using a Retcam II fundoscopy system (Clarity Medical Systems) followed by, only in rabbits, ultrasound imaging using an ultrasound biomicroscope (UBM Plus, Accutome, Malvern, PA, USA) to assess injection success and visualize bleb formation.

### 5.5 Electroretinogram

ERG was performed in rats. Animals were dark adapted overnight. Whole body anesthesia was induced by intraperitoneal injection of ketamine:xylazine (80 mg/kg:8 mg/kg) followed by topical anesthesia (0.5% proparacaine hydrochloride ophthalmic solution, Akorn) and pupil dilation (1% tropicamide ophthalmic solution, Henry Schein). Full‐field scotopic ERG responses were recorded at stimulus intensities of 0.001, 0.005, 0.01, 0.1, 1 and 10 cd s/m^2^ using a Diagnosys Celeris system (Diagnosys, Lowell, MA, USA). A‐, b‐, and c‐waves were subsequently extracted. Animals were kept on a heating pad for the duration of ERG collection and after recovery from anesthesia.

### 5.6 Histology

Eyes were immediately placed in 20 mL fixative solution chilled in dry ice containing 97% methanol (Sigma‐Aldrich, St. Louis, MO USA) and 3% acetic acid (Thermo Fischer Scientific) upon enucleation and stored at −80°C. After 10 days, eyes were left at room temperature (20–25°C) for 2 h to warm up, followed by removal of cornea and lens using a razor blade. Samples were then serially immersed in 100% ethanol (reagent grade alcohol, VWR, Radnor, PA, USA) repeated twice and then in xylene (VWR) repeated twice each time for 2 h, followed by incubation in a first paraffin bath for 4 h and a second paraffin bath overnight. Eyes were then embedded in paraffin blocks, sectioned by 7‐μm thick cuts using a rotary microtome (HM 355 S, Thermo Fischer Scientific). Some sections were stained with H&E using an autostainer instrument (Autostainer XL, Leica, Deer Park, IL, USA). Sections were finally imaged using a brightfield microscope (Axiocam 506 color, Carl Zeiss Microscopy, White Plains, NY, USA).

### 5.7 ONL cell nuclei count

Analysis was performed on H&E‐stained histological sections of the injected eyes collected at different timepoints of 24 h (*n* = 3), 72 h (*n* = 3), 10 days (*n* = 3), and 6 weeks (*n* = 3), and control eyes (*n* = 3). For each eye, one histological section from the bleb region that included the MN puncture site was selected. Cell nuclei were counted using Adobe Photoshop count tool (Adobe Systems, San Jose, CA, USA) in increments of 100 μm‐wide segments extending 2.1 mm posteriorly from the far retinal periphery. The first 100 μm segment of retinal periphery was excluded in all eyes.

### 5.8 Immunohistochemistry

#### 5.8.1 Lectin and anti‐IBA1 staining

All staining was performed using a Sequenza staining system (Epredia, Kalamazoo, MI, USA). Upon deparaffinization and rehydration, slides were mounted on Sequenza coverplate. A volume of 400 μL of blocking buffer containing 2.5% normal donkey serum in Tris‐buffered saline (TBS, 46‐012‐CM, Corning, Corning, NY, USA) were added to the slide and incubated at room temperature for 30 min. A volume of 130 μL of the primary antibody solution containing 1:200 rabbit anti‐IBA1 (ab178847, Abcam, Waltham, MA, USA) in blocking buffer was added to the slide and incubated at room temperature for 1 h followed by two washes with 1 mL TBST, 5 min each. Then, 200 μL of the secondary antibody solution containing 1:1000 donkey anti‐rabbit.IgG‐AF647 (A32795, Life Technologies, Waltham, MA USA) in blocking buffer were added to the slide and incubated at room temperature for 1 h and then washed twice with 1 mL TBS containing 0.1% Tween‐20 (TBST), 5 min per wash. Slides were then stained with 1:200 dilution of Tomato lectin conjugated to fluorescein (Vector Labs FL‐1171‐1, Newark, CA, USA). The slides were then washed with 1 mL of TBS for 5 min, counterstained with 400 μL of 2.5 uM Hoechst 33342 for 10 min, washed with 5 mL TBS for 5 min, mounted, and coverslipped using Vectashield Vibrance mountant (Vector Labs H‐1700, Newark, CA, USA). Slides were finally stored at room temperature in a dark box until imaging by confocal microscopy (Nikon instruments, Melville, NY, USA).

#### 5.8.2 RPE65 and Rhodopsin staining

Upon deparaffinization and rehydration, slides were mounted on Sequenza coverplate as described above. A volume of 400 μL of blocking buffer containing 2.5% normal donkey serum in TBS were added to the slide and incubated at room temperature for 30 min. A volume of 130 μL of the primary antibody solution containing 1:200 mouse anti‐rhodopsin (sc‐57433 AF647, Santa Cruz Biotechnology, Dallas, TX, USA) and 1:1000 rabbit anti‐RPE65 (gift from John Nickerson) in blocking buffer was added to the slide and incubated at room temperature for 1 h followed by two washes with 1 mL TBST, 5 min each. Then, 200 μL of the secondary antibody solution containing 1:1000 donkey anti‐rabbit.IgG‐AF488 (A32790, Life Technologies, Waltham, MA USA) in blocking buffer were added to the slide and incubated at room temperature for 1 h and then washed twice with 1 mL TBST (5 min per wash). The slides were then counterstained with 400 μL of 2.5 μM Hoechst 33342 for 10 min, washed with 5 mL TBS for 5 min, mounted and coverslipped using Vectashield Vibrance mountant (Vector Labs H‐1700, Newark, CA, USA), and imaged as described above.

#### 5.8.3 Anti‐GFP staining

Following a procedure similar to IBA1 staining above, slides were stained using a primary antibody solution containing 1:200 anti‐GFP antibody conjugated to AlexaFluor 647 (sc‐9996 AF647, Santa Cruz Biotechnology, Dallas, TX, USA) in blocking buffer without a secondary antibody staining step.

#### 5.8.4 TUNEL staining

Staining was performed using Promega DeadEnd TUNEL Fluorometric kit (Product #G3250, Promega, Madison, WI, USA). Briefly, after deparaffinization and rehydration, slides were mounted in the Sequenza system. Two‐hundred microliter of Z‐fix solution (Anatech, Battle Creek, MI, USA) were added to each slide and incubated for 15 min. After washing twice with 1 mL of PBS, slides were then incubated in 200 μL of Proteinase K for 8 min and washed with 1 mL of PBS for 5 min. After incubation in 200 of Z‐fix solution for 5 min and washing with PBS, slides were subsequently immersed for 2 h in 130 μL of labeling reaction mix consisting of 124 μL equilibration buffer, 1 μL rTDT enzyme and 5 μL nucleotide mix. Lastly, slides were washed with TBS, counterstained, and coverslipped following the procedure described above.

### 5.9 RPE flatmount preparation and immunofluorescence staining

Control (*n* = 3) and injected (*n*= 4) eyes were enucleated from rats upon euthanasia 6 weeks after subretinal injection, immediately fixed in Z‐fix (Anatech, Battle Creek, MI, USA) for 18 min and rinsed three times using HBSS (Mediatech, Manassas, VA, USA). Under a dissecting microscope, excess extraocular tissue was removed, the center of the cornea was punctured using a razor blade, and four radial cuts were made from the corneal puncture extending back towards the optic nerve. Lens, iris, vitreous, and retina were removed by forceps and ocular flaps were flattened with RPE monolayer facing up.

For immunofluorescence staining, flatmounts were blocked in 0.1% Triton X‐100 + 1% bovine serum albumin in HBSS blocking buffer for 1 h at room temperature followed by immunostaining with anti‐zonula occludens‐1 (ZO‐1, sc‐33725 AF488, Santa Cruz Biotechnology) at 1:100 dilution in blocking buffer overnight at 4°C. Flatmounts were then washed five times for 2 min each with HBSS containing 0.1% Triton X‐100. For nuclei staining, samples were washed with 2.5 μM Hoechst 33342 (1:250) in blocking buffer for 10 min and finally washed twice for 2 min each with HBSS containing 0.1% Triton X‐100 before being mounted and coverslipped using Vectashield Vibrance. All solutions contained 0.01% sodium azide. Flatmounts were then imaged using a Nikon Ti2 inverted microscope with an A1R confocal scanner (Nikon instruments).

### 5.10 RPE flatmount image segmentation

Three square, 700 μm × 700 μm, images were sampled from the entire RPE flatmount images of the injected eyes to represent RPE at three zones: MN injection site, bleb region and an area outside the bleb. For control eyes, one 700 μm × 700 μm image was obtained. All images were selected 1–2 mm from far retinal periphery. Selected images were then segmented using an image processing program (Imaris Version 9, Oxford instruments, Zurich, Switzerland). Cell borders were automatically identified and morphometric data including cell area and cell count were extracted for each image.

### 5.11 Statistical analyses

Statistical analyses were performed via GraphPad Prism 9.3.1 (GraphPad Software, San Diego, CA, USA). Hemorrhage survival rates after trans‐choroidal insertion of different needle sizes were compared using a Log‐rank (Mantel‐Cox) test. For the ONL cell count study, a two‐way ANOVA (on the factors of timepoints and retinal location) with Sidak's correction for multiple comparisons was performed. Differences between the ERG amplitudes were assessed using a paired *t*‐test. Only the highest amplitudes (step 6) were compared. Data are reported as mean ± standard deviation and *p* values less than 0.05 were considered statistically significant in all tests. Ability to perform subretinal delivery by trans‐scleral MN injection was tested in a large cohort of *n* = 65 rat eyes with proof‐of‐concept trials in *n* = 7 mice eyes, *n* = 4 guinea pig eyes, and *n* = 3 rabbit eyes. Incidence of ocular bleeding following trans‐scleral MN injection was similarly tested in a cohort of *n* = 31 injected rat eyes. Other studies (e.g., safety examinations) were powered by up to *n* = 10 samples with at least *n* = 3 eyes per experimental groups to enable statistical analysis.

## AUTHOR CONTRIBUTIONS

**Amir Hejri:** Conceptualization; investigation; writing – original draft; methodology; validation; visualization; writing – review and editing; project administration; formal analysis. **Micah A. Chrenek:** Investigation; writing – review and editing. **Nolan T. Goehring:** Investigation; writing – review and editing. **Isabella I. Bowland:** Investigation; writing – review and editing. **Richard Noel:** Investigation; writing – review and editing. **Jiong Yan:** Methodology; writing – review and editing. **John M. Nickerson:** Writing – review and editing; supervision; methodology. **Mark R. Prausnitz:** Supervision; funding acquisition; writing – review and editing; conceptualization; methodology; visualization.

## FUNDING INFORMATION

This work was supported by a Challenge Grant from Research to Prevent Blindness, Inc. to the Ophthalmology Department at Emory University and National Institutes of Health (NIH) grants R01EY028450, R01EY021592 and P30EY006360.

## CONFLICT OF INTEREST STATEMENT

MRP is an inventor of patents, consultant to companies, and co‐founder/shareholder of companies in the field of microneedle technology. AH, JY, JMN, and MRP are inventors on patent applications on the subject of this study. All other authors declare they have no competing interests.

## ETHICS STATEMENT

We support the ethics and integrity policies of the journal and have therefore provided in the manuscript: data availability statement, funding statement, conflict of interest disclosure, and ethics approval statement for animal studies.

## Supporting information


**Data S1.** Supporting information.

## Data Availability

The data that support the findings of this study are available in the article text and figures, and in the Supplementary Material of this article.
