# Supplementary material for: A non‐surgical method for subretinal delivery by trans‐scleral microneedle injection
Source: Bioeng Transl Med. 2025 Jan 28;10(3):e10755. doi: 10.1002/btm2.10755 (PMC12079348; doi:10.1002/btm2.10755)
Supplement: Supplementary file 1 — Data S1. Supporting information. [file BTM2-10-e10755-s001.docx]

**Supplementary Materials**

**A non-surgical method for subretinal delivery by trans-scleral microneedle injection**

Amir Hejri, Micah A. Chrenek, Nolan T. Goehring, Isabella I. Bowland, Richard Noel, Jiong Yan, John M. Nickerson, Mark R. Prausnitz

**Table S1. Determination of microneedle (MN) length for subretinal injection**

|  | Mouse | Rat | Guinea Pig | Rabbit |
| --- | --- | --- | --- | --- |
| Optimal MN length for SCS injection^1^ | ~60 µm | 160 µm | 260 µm | 750 µm |
| Thickness of choroid + RPE^2^ | ~50 - 60 µm | | | ~350 µm |
| MN length used for subretinal injection^3^ | 120 µm | 220 µm | 300 µm | 1100 µm |

^1^These values were previously determined and reported in reference [1] for rats and guinea pigs and in reference [2] for rabbits. The value for mice indicates the approximate thickness of sclera, since SCS injections were not performed in mice. ^2^These values were estimated from references [3-6] for rodents. The value for rabbit assumes similar choroidal thickness as the human eye [7]. ^3^This value was informed by the sum of the two numbers in the rows above.


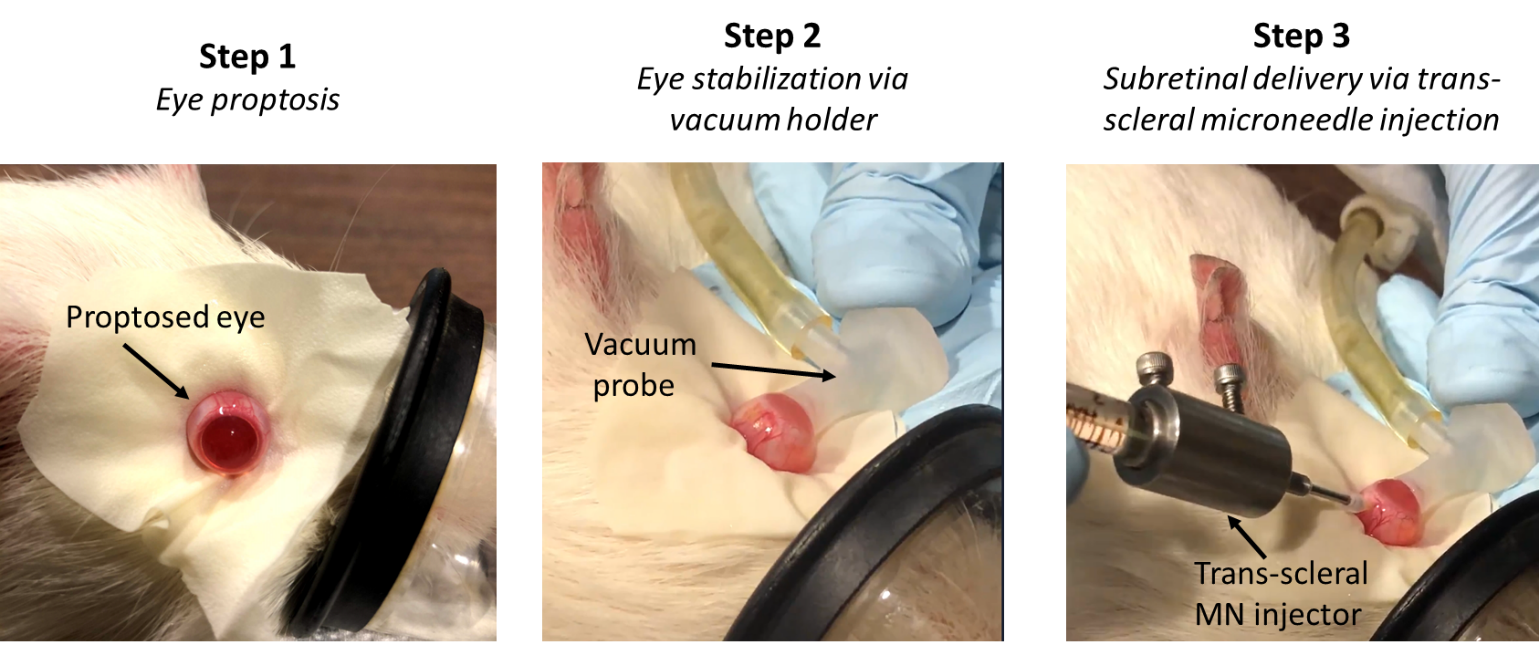


**Figure S1.** Trans-scleral subretinal injection technique using microneedle insertion. Representative image of subretinal delivery in a guinea pig eye in vivo


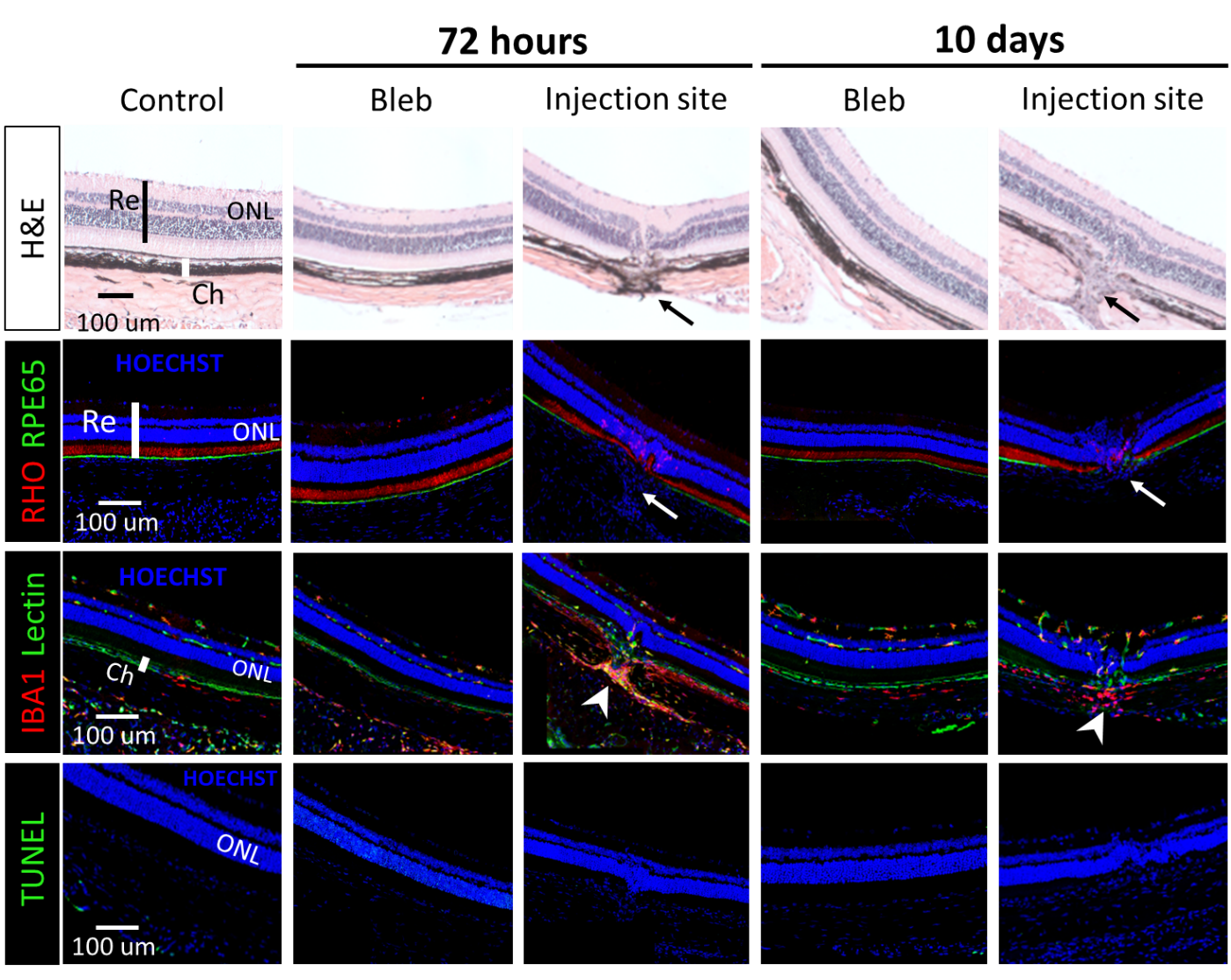


**Figure S2**. Post-mortem safety analysis 72 hours and 10 days after subretinal delivery of HBSS via trans-scleral MN injection in rats in vivo. The microscopic puncture of choroid and RPE (**Fig. 5**) did not expand over time as seen in hematoxylin and eosin (H&E) stained images. Similarly, focal loss of RPE and photoreceptor outer segment observed at 24 h (**Fig. 5**) remained limited to the puncture site and did not grow over time, as shown by RPE65 and RHO antibody staining, respectively. Mild macrophage reaction seen at 24 h (**Fig. 5**) persisted up to 10 days, as shown by IBA1 staining. No evidence of neovascularization or apoptotic cell death was found shown by GS Lectin and TUNEL staining, respectively. Nothing notable was observed in the bleb regions. The study was conducted in N=17 rat eyes including N=3 eyes examined at 24 hours (**Fig. 4**), N=6 eyes at 6 weeks (**Fig. 4**), N=4 at 72 hours and N=4 at 10 days timepoint. Contralateral eyes were used as controls.


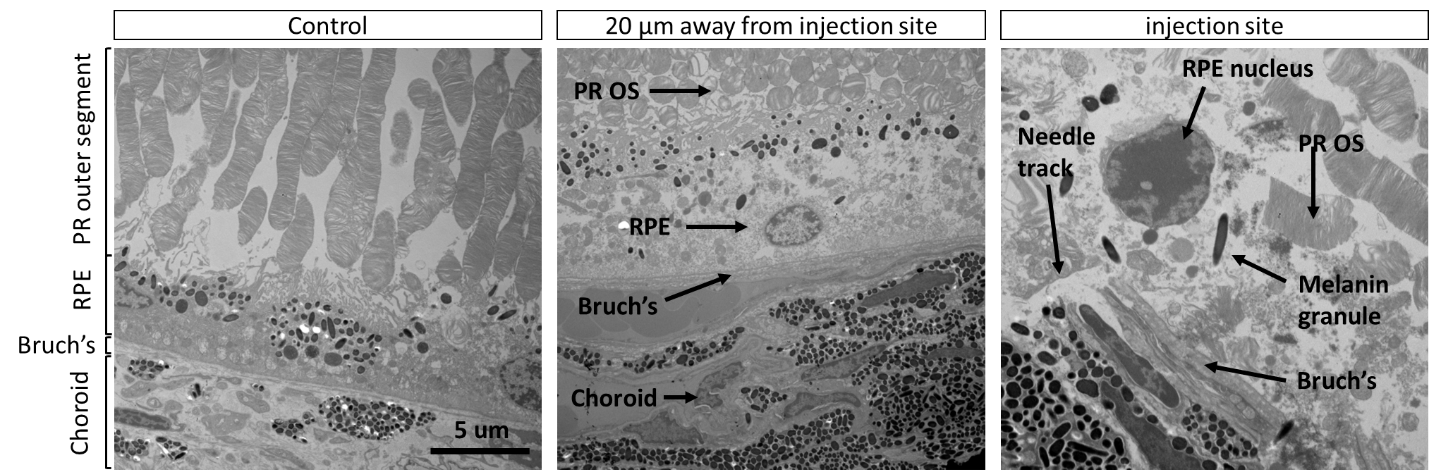


**Figure S3**. Transmission electron microscopy images of ocular sections six weeks after subretinal delivery of HBSS via trans-scleral MN injection in rat in vivo. Evidence of penetration across choroid, Bruch’s membrane and RPE is seen at the puncture site. This effect is highly localized and normal tissue morphology resumes immediately next to the injection site as close as 20 µm away, indicating no retinal puncture and no damage away from the needle track. Imaging was performed in N=1 eye.


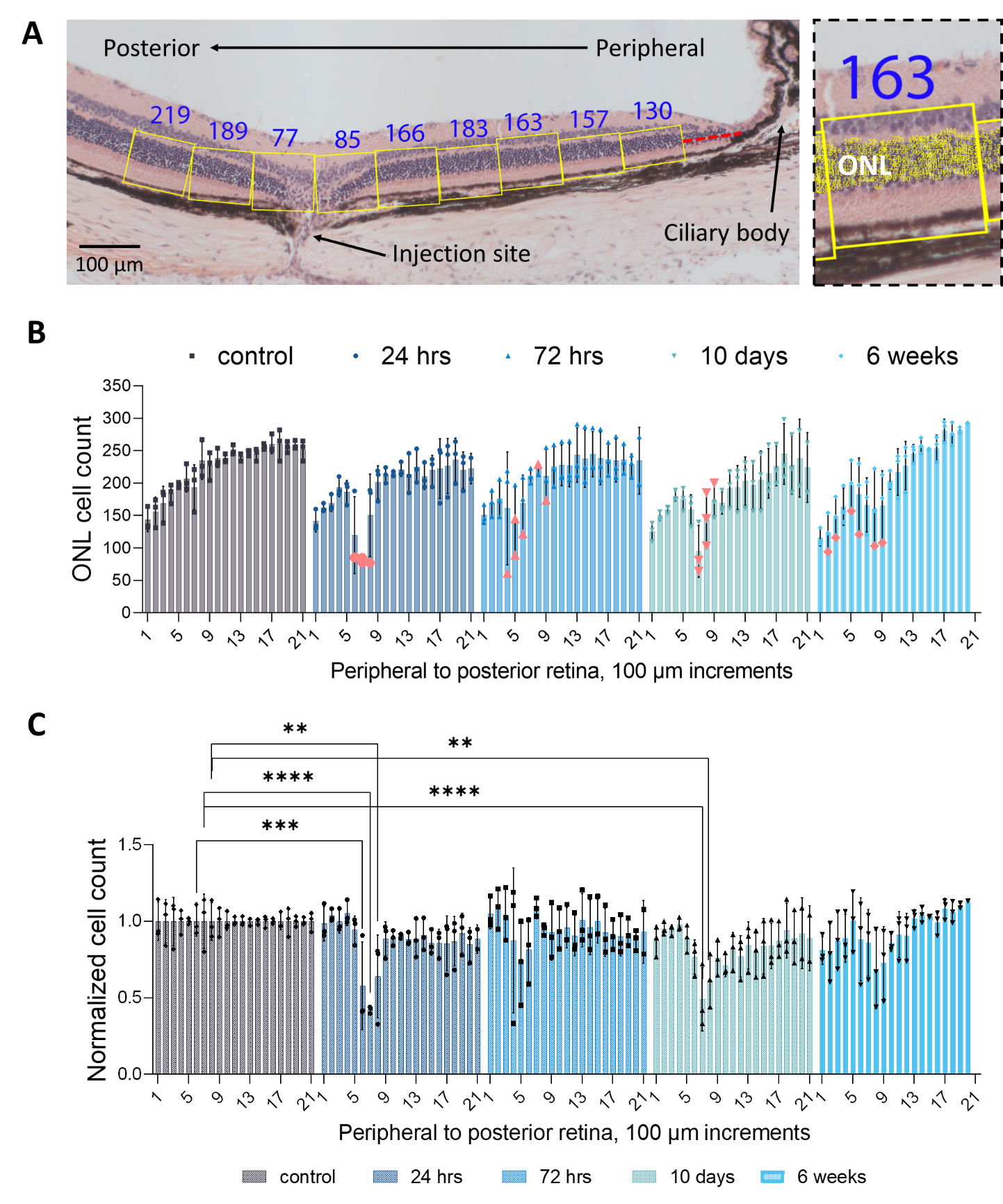


**Figure S4**. Analysis of outer nuclear layer (ONL) cell count after subretinal delivery of HBSS via trans-scleral MN injection in rats in vivo. (A) The number of cell nuclei of photoreceptor cells in the ONL was measured in 100 µm increments starting with peripheral retina near ciliary body (sparing the first 100 µm, dashed red line) to 2.1 mm posteriorly. Numbers indicate cell count in each box. Inset provides a magnified view. (B) Results of cell count measurements at various timepoints after subretinal injection. Data were collected from a histological tissue section from the bleb region that included puncture site (n=3 eyes per timepoint). Red data points correspond to cell counts in the puncture site area and show highly localized photoreceptor cells loss at the puncture site of some eyes. Control data demonstrate a characteristic increasing cell count, moving posteriorly reflecting a thicker posterior in normal retina. (C) Cell counts normalized by the average value of each bar in the control eye reflecting no cell loss in the bleb region other than at the puncture site in some eyes (24 h and 10 days). Significant differences are shown: *p < 0.05, **p < 0.01, ***p < 0.001, ****p < 0.0001. Study groups included N=3 rat eyes at 24 hours, N=3 eyes at 72 hours, N=3 eyes at 10 days, N=3 eyes at 6 weeks and N=3 control eyes. Statistical analysis was performed using a two-way ANOVA (on the factors of timepoints and retinal location) with Sidak’s correction for multiple comparisons.


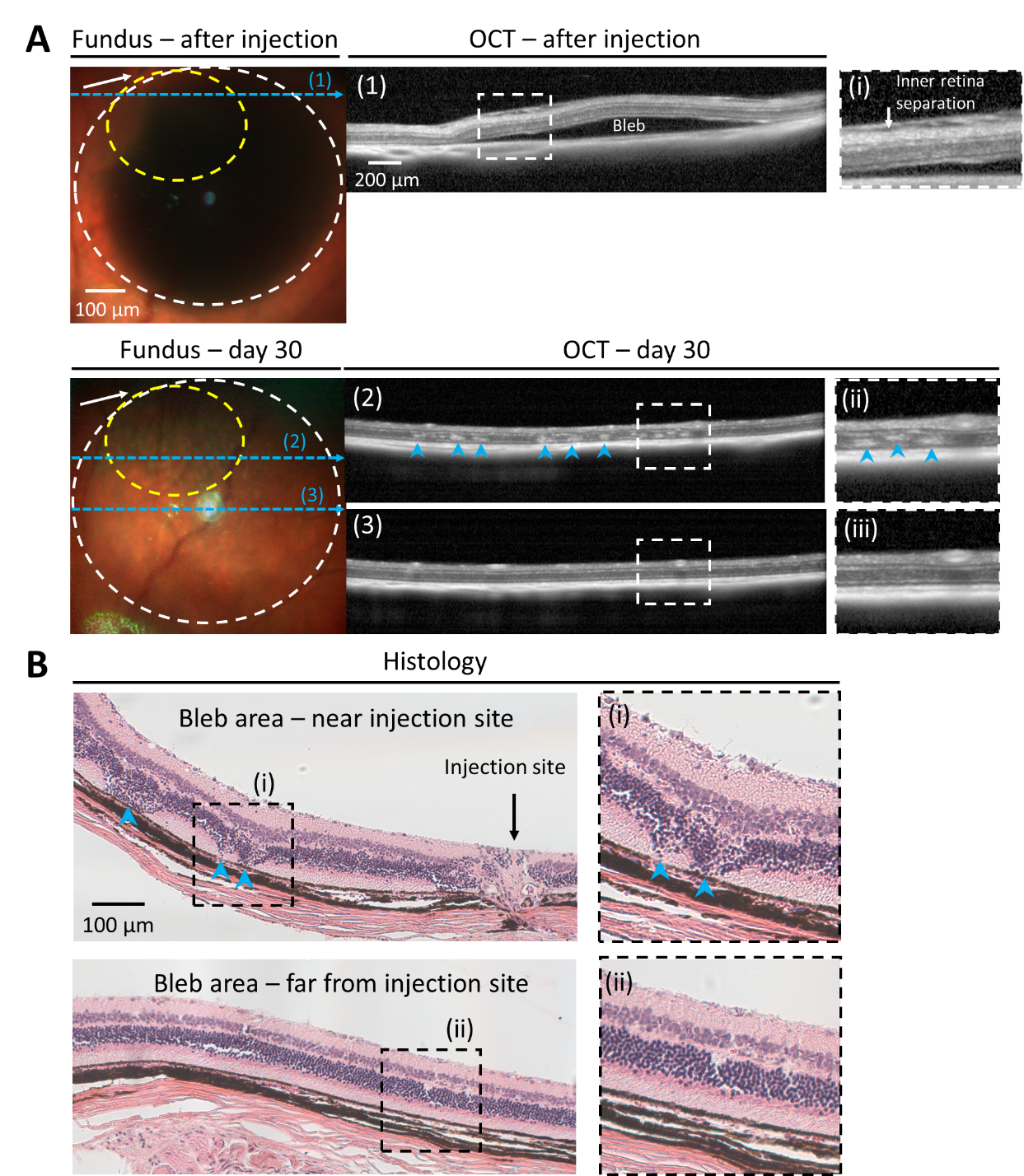


**Figure S5**. Evidence of injection into inner retinal layers after subretinal delivery of HBSS via trans-scleral MN injection in rats in vivo. (A) Representative fundus and optical coherence tomography (OCT) images showing subretinal bleb formation (dashed white circles, fundus) with limited injection into inner retinal layers near injection site (dashed yellow circle, fundus). Inset (i) provides a magnified view indicating expansion of inner retinal layers following injection. Retinal abnormalities (blue arrowheads, OCT) emerge 30 days post-injection near the injection site (OCT, 2) corresponding to the area of inner retinal delivery. Retina appears normal outside the inner retinal delivery region (OCT, 3). Insets (ii and iii) provide magnified views. OCT images correspond to the sites of the dashed blue lines in fundus images. (B) Representative histological tissue sections indicating retinal damage, including outer nuclear layer disorganization and focal loss of photoreceptor outer segments (blue arrowheads) near the injection site. Retina in the bleb region far from the injection site appears normal. Retinal damage is likely associated with expansion of the inner retina. Delivery into the inner retinal layers may have been caused by penetration of the fluid jet stream exiting the MN tip into soft retinal layers. The study included N=4 eyes.


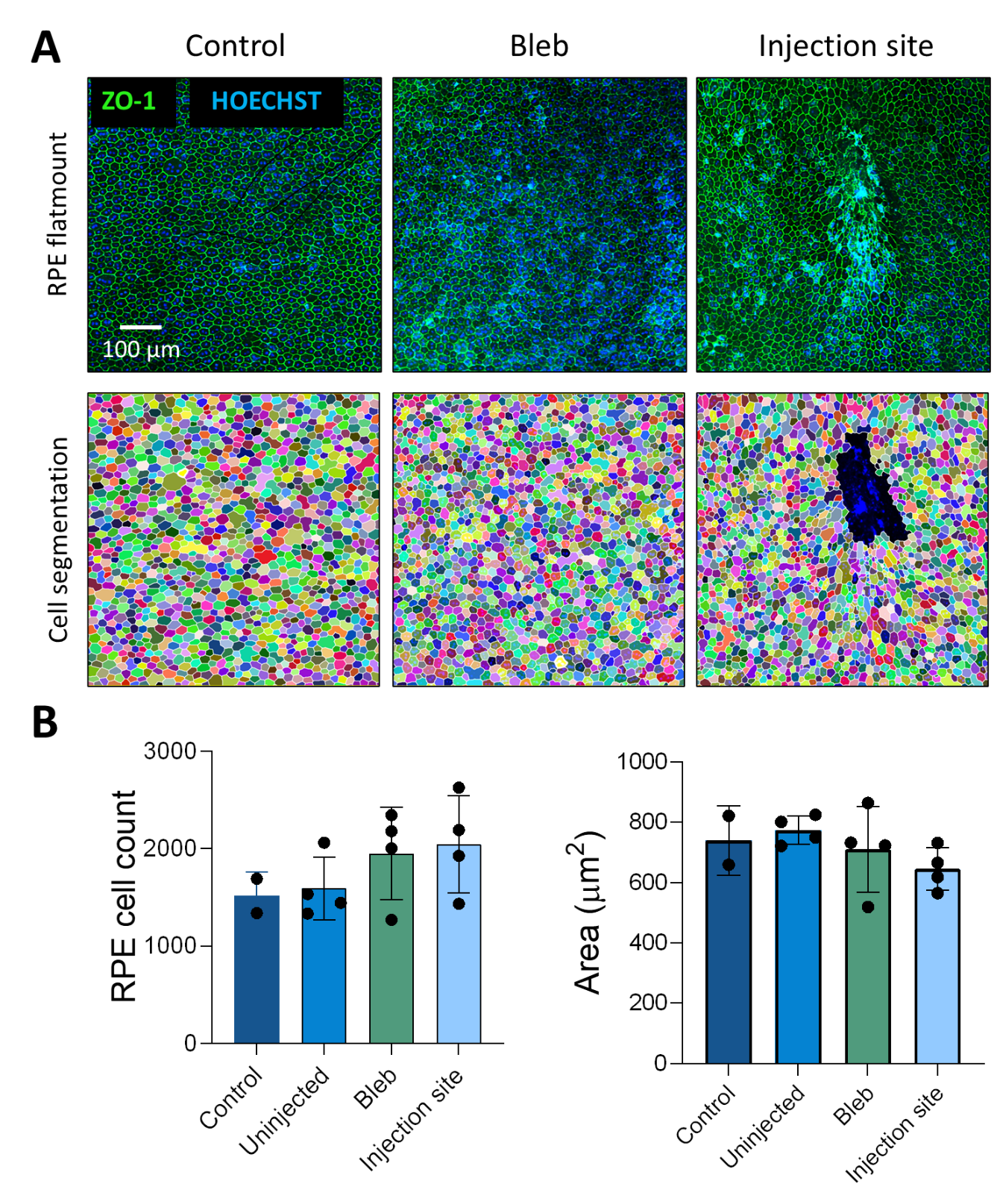


**Figure S6**. Morphometrical analysis of retinal pigment epithelium (RPE) flatmount six weeks after subretinal delivery of HBSS via trans-scleral MN injection in rats in vivo. (A) Representative flatmount confocal microscopy images of RPE monolayer in 700 µm X 700 µm areas at the injection site, bleb, and naïve control monolayer, and corresponding cell segmented data after image processing. A small area in the injection site image, with an average size of 0.027 ± 0.012 mm^2^, could not be segmented, indicating cell loss due to MN puncture. (B) Numerical morphometric data extracted from segmented images indicating no statistically significant (p>0.05) alterations in RPE cell count and individual cell area at the injection site, bleb area and uninjected area (outside bleb region) versus naïve control eye. Notably, average cell count did not change in the injection site area in spite of RPE cell loss, averaging 36 ± 17 cells, at the puncture site, implying minimal tissue damage. The study included N=4 injected eyes and N=3 contralateral eyes as controls. Statistical analysis was done using a paired t-test.

**
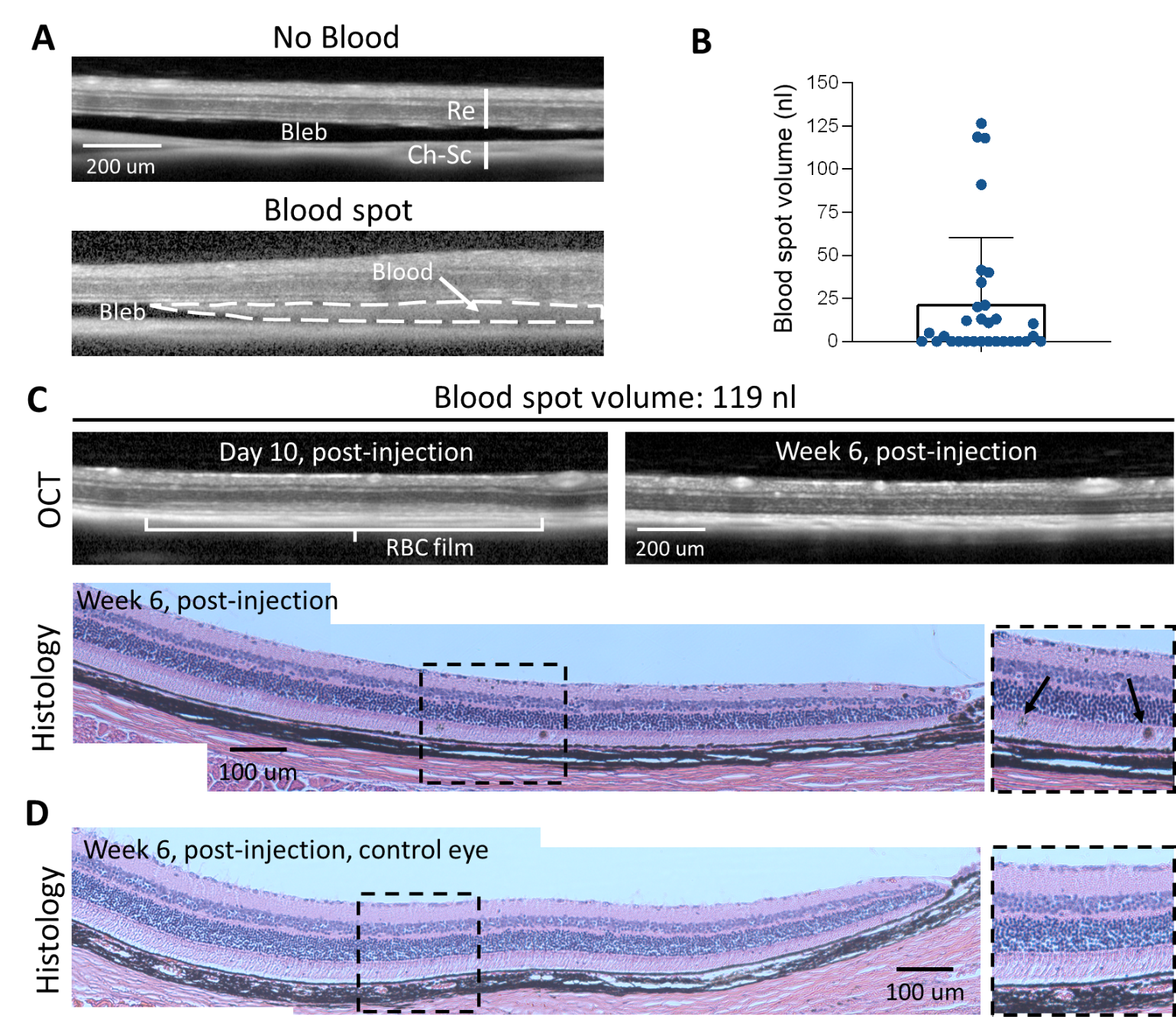
**

**Figure S7.** Incidence and characterization of highly localized, microscopic blood spot and its effect on retinal morphology after subretinal delivery of HBSS using trans-scleral MN injection in rat in vivo. (A) Representative cross-sectional OCT images taken immediately after injection showing bleb appearance with or without subretinal blood spot. (B) Assessment of possible subretinal bleeding in 31 eyes injected with 1-3 µl HBSS. Blood spot volume was calculated from serial OCT images that cover the bleb area and were taken immediately after injection. (C) Representative OCT and histological examination of an eye with the largest blood spot showing a transient red blood cell film in the subretinal space on day 10 which self-resolved by week six post injection. Histology of this eye showed normal retinal morphology except for rare pigment granules in subretinal space (arrows). Inset provides a magnified view. (D) Histological tissue section of a naive control eye for comparison. OCT: optical coherence tomography, Re: retina, Ch-Sc: choroid-sclera. The study included N=31 eyes. Additional images and analysis of ocular blood spots are shown in **Fig. S8** below.


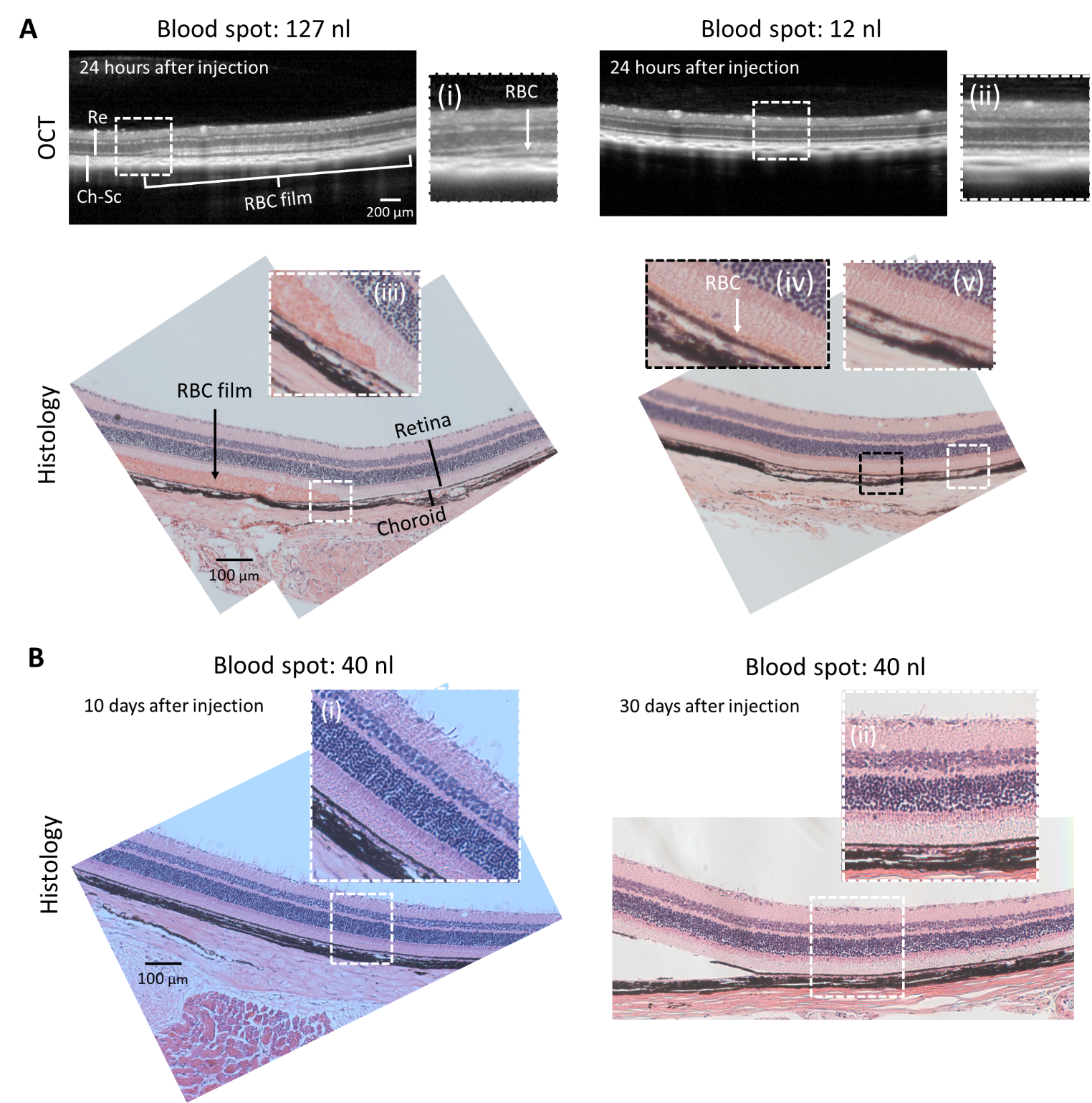


**Figure S8**. Observations related to subretinal blood spot and retinal morphology after subretinal delivery of HBSS via trans-scleral MN injection in rats in vivo. (A) Representative OCT and histological tissue sections comparing ocular morphology 24 h post-injection in eyes with the highest (127 nl) versus less-than-average (12 nl) blood spot. A layer of red blood cell (RBC) deposition was seen in the SRS of the eye with the larger blood spot. Insets (i) and (iii) provide magnified views. A RBC film was not found in the OCT image of the eye with the small blood spot (inset ii) with very few individual RBCs seen in the histology section, that could only be seen upon magnification and existed in a small, limited area (inset iv) with neighboring retina appearing normal (inset v). (B) Representative histological tissue section images of eyes with greater-than-average blood spot (40 nl), indicating no RBCs in the SRS and healthy retina on days 10 and 30 post-injection. Insets provide magnified views. OCT: optical coherence tomography. The study included N=31 eyes.


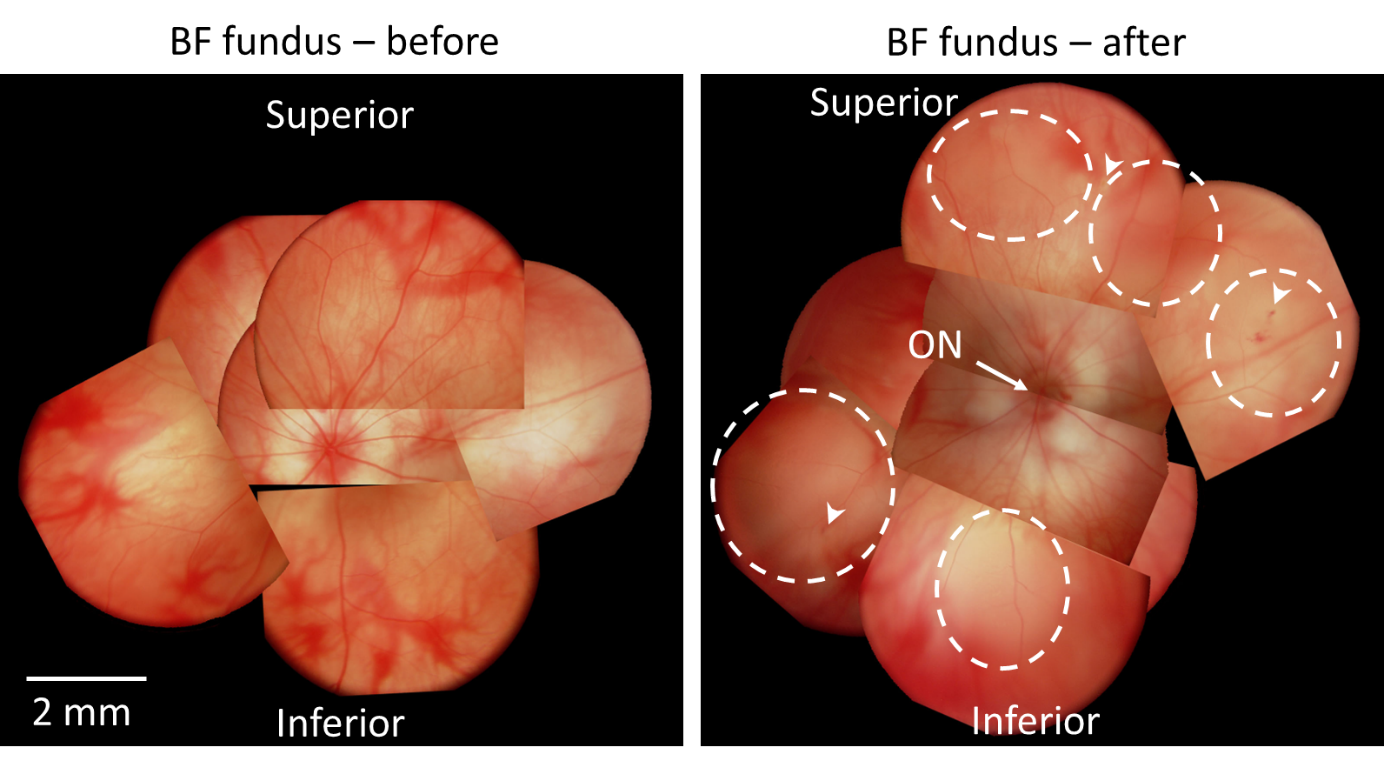


**Figure S9.** Brightfield (BF) fundus images taken before and after five sequential subretinal injections of HBSS using a trans-scleral MN in a rat eye in vivo. No evidence of intraocular or extraocular bleeding was seen. Dashed circles mark the location of subretinal blebs. Arrowheads indicate injection sites in three injections. Complete rendering of rat fundus was created by stitching together images captured at different locations on the fundus. ON: optic nerve. The study included N=1 eye.


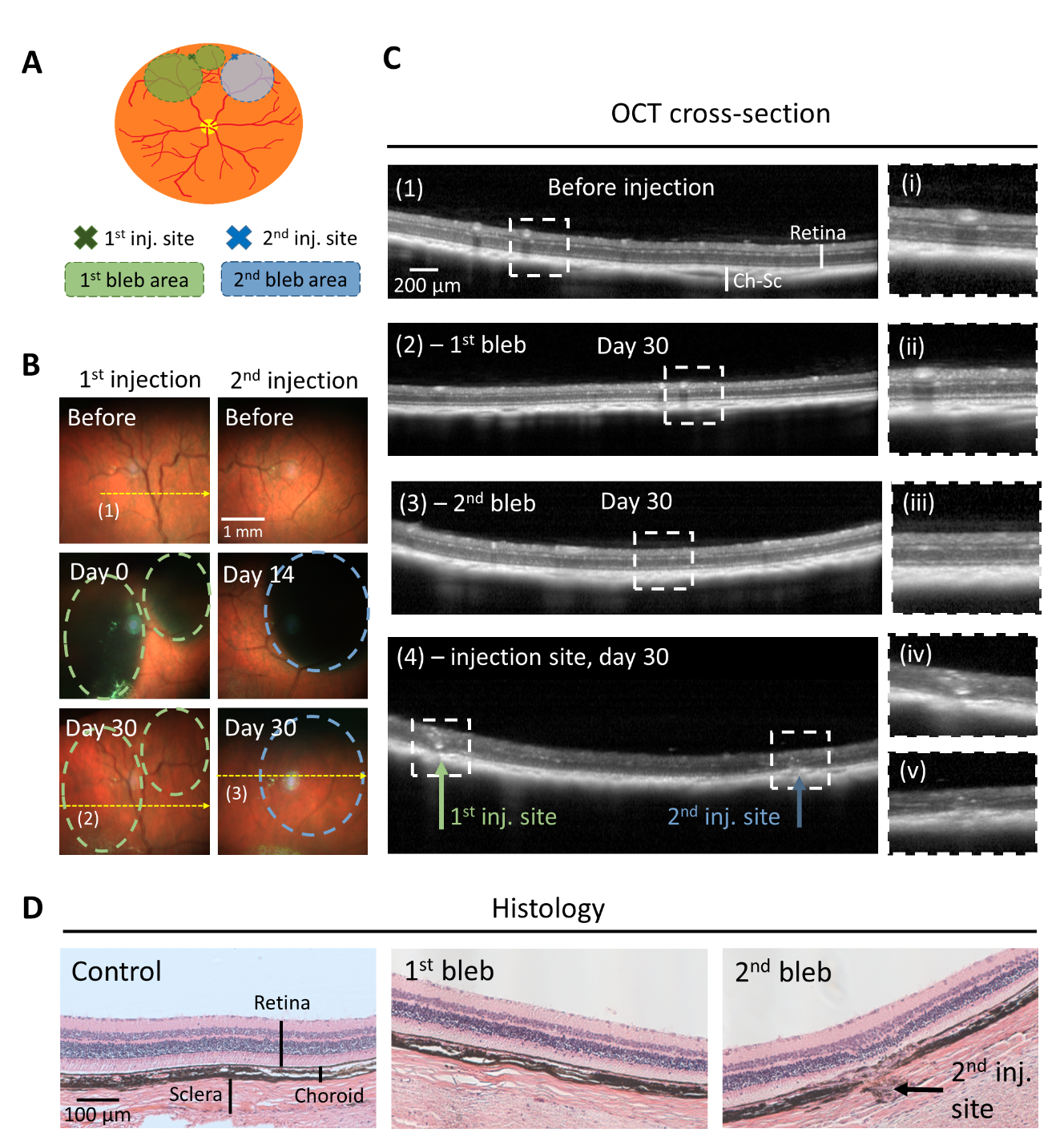


**Figure S10**. Subretinal delivery of HBSS via sequential trans-scleral MN injections in rat in vivo. (A) An illustration depicting the fundus of an eye that received two subretinal injections, 14 days apart, where the two blebs did not overlap. (B) Representative brightfield fundus images taken at various timepoints up to 30 days after the first injection. Dashed green and blue circles indicate bleb areas after first and second injection, respectively. (C) Representative optical coherence tomography (OCT) images taken before (1) and 30 days after injection, highlighting ocular structure in the first (2) and second bleb (3) areas. OCT (4) highlights the injection sites. OCT images (1-3) correspond to the sites of the dashed yellow lines in the fundus images in (B). Retina appears with no notable abnormalities. Insets provide magnified views. (D) Representative histological tissue sections obtained upon termination of the study on day 30. Nothing notable was observed in the bleb regions of both injections, other than evidence of MN puncture across choroid and RPE at the injection site. Eyes received subretinal administration of 3 µl HBSS solution in both injections. The study included N=10 eyes.


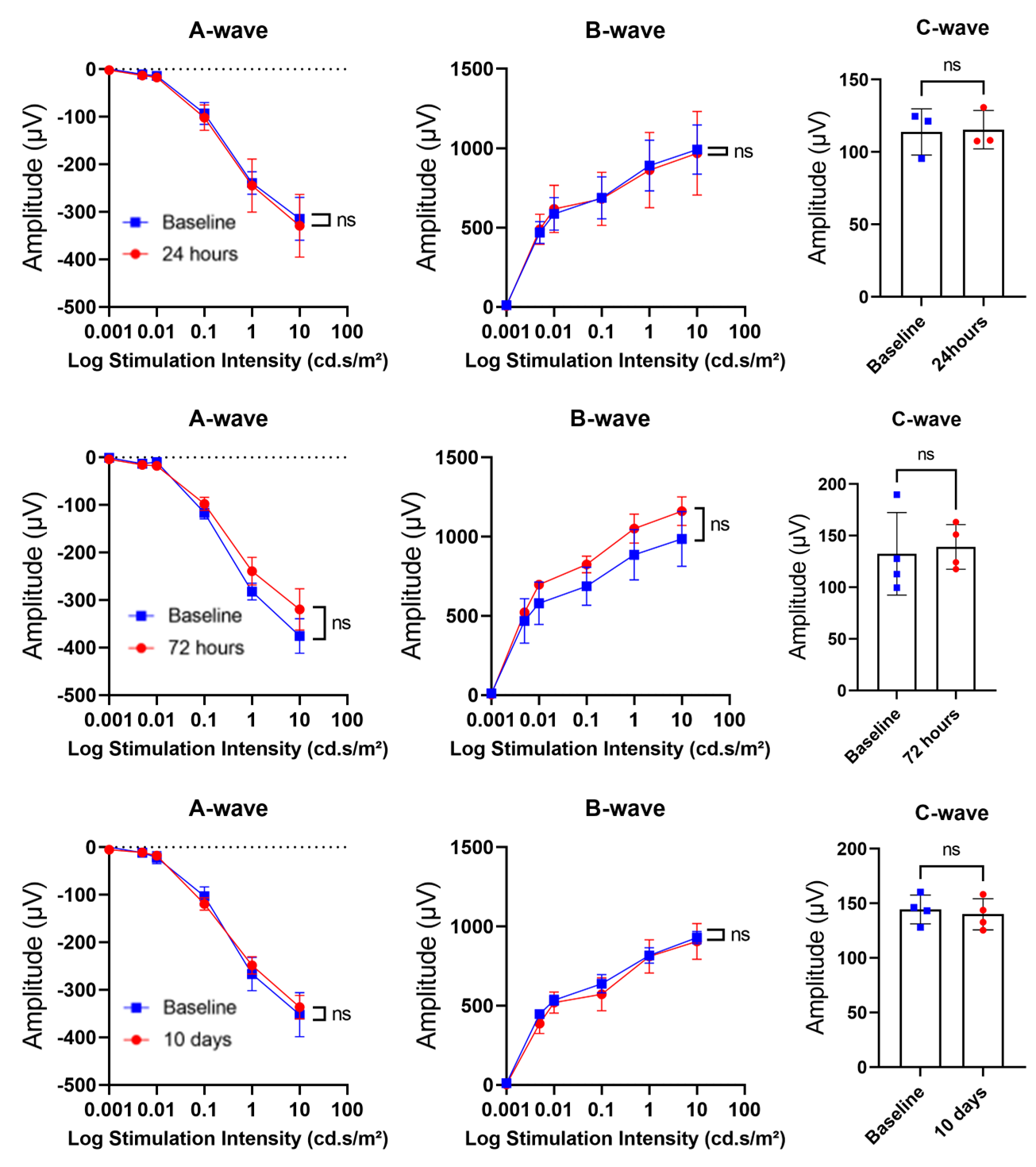


**Figure S11**. Electroretinogram (ERG) responses collected 24 h (N=3 eyes), 72 h (N=4 eyes) and 10 days (N=4 eyes) after subretinal delivery of HBSS via a single trans-scleral MN injection in rats in vivo, indicating no reduction in ERG wave amplitudes. A-wave, B-wave and C-wave indicate photoreceptor cell, bipolar cell and retinal pigment epithelium (RPE) cell responses, respectively. Differences between the ERG amplitudes were assessed using a paired t-test (only the highest amplitudes were compared).


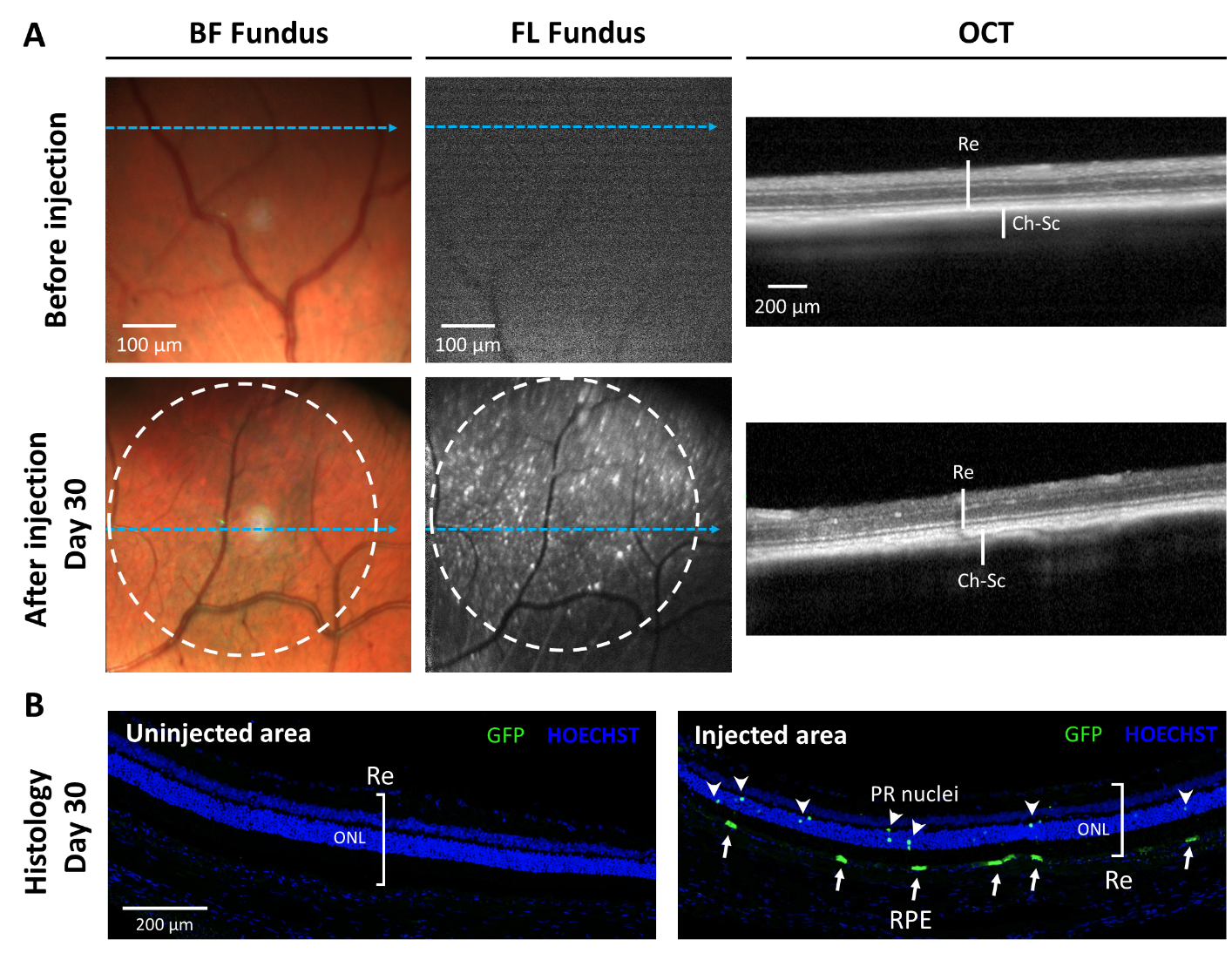


**Figure S12.** Subretinal delivery of AAV2 vectors via trans-scleral MN injection in rat in vivo. (A) Representative fundus and OCT images taken before and 30 days after injection of 1 µl HBSS containing AAV2-CMV-GFP vectors at a titer of 1.8 × 10^13^ viral genomes/ml (N=4). Green fluorescent protein (GFP) expression is evident in the injected area (dashed circle) in fluorescence (FL) fundoscopy images taken on day 30 post-injection. Brightfield (BF) fundus and optical coherence tomography (OCT) images show no evidence of retinal abnormalities up to 30 days. (B) Representative histological sections stained with anti-GFP antibody and counterstained with Hoechst to visualize cell nuclei. Arrows and arrowheads indicate GFP expression in some retina pigment epithelium (RPE) and photoreceptor (PR) cells, respectively. Lack of widespread GFP expression may be due to low viral vector titer used in injections. OCT images correspond to the dashed blue arrow in fundus images. ONL: outer nuclear layer. Re: retina, Ch-Sc: choroid-sclera. The study included N=8 eyes.

REFERENCES

1. Hejri, A., et al., *Suprachoroidal Delivery in Rats and Guinea Pigs Using a High-Precision Microneedle Injector.* Translational Vision Science & Technology, 2023. **12**(3): p. 31-31.

2. Chae, J.J., et al., *Drug‐Free, Nonsurgical Reduction of Intraocular Pressure for Four Months after Suprachoroidal Injection of Hyaluronic Acid Hydrogel.* Advanced Science, 2021. **8**(2): p. 2001908.

3. El-Nimri, N.W., et al., *Effect of topical latanoprost on choroidal thickness and vessel area in Guinea pigs.* Experimental Eye Research, 2022. **225**: p. 109286.

4. Jnawali, A., K.M. Beach, and L.A. Ostrin, *In vivo imaging of the retina, choroid, and optic nerve head in guinea pigs.* Current eye research, 2018. **43**(8): p. 1006-1018.

5. Campos, A., et al., *Choroidal and retinal structural, cellular and vascular changes in a rat model of type 2 diabetes.* Biomedicine & Pharmacotherapy, 2020. **132**: p. 110811.

6. Berkowitz, B.A., et al., *Oxidative stress and light-evoked responses of the posterior segment in a mouse model of diabetic retinopathy.* Investigative ophthalmology & visual science, 2015. **56**(1): p. 606-615.

7. Brown, J.S., et al., *In vivo human choroidal thickness measurements: evidence for diurnal fluctuations.* Investigative ophthalmology & visual science, 2009. **50**(1): p. 5-12.
